# Integrating Morphology and Chloroplast Genomics: A New East Asian Species of *Aletris* (Nartheciaceae) With Insights Into Regional Phylogeny and Evolution

**DOI:** 10.1002/ece3.72654

**Published:** 2026-01-05

**Authors:** Xiong Li, Yong‐Ling Qiu, Jiang‐Tao Li, Bo Xu, Qi Yu, Wen‐Bin Ju

**Affiliations:** ^1^ Mountain Ecological Restoration and Biodiversity Conservation Key Laboratory of Sichuan Province. Chengdu Institute of Biology Chinese Academy of Sciences Chengdu China; ^2^ College of Life Sciences University of Chinese Academy of Sciences Beijing China; ^3^ Wild Plants Sharing and Service Platform of Sichuan Province Chengdu China

**Keywords:** *Aletris medogensis*, comparative chloroplast genome analysis, East Asia, IR boundary dynamics, molecular markers, Nartheciaceae, selective pressure

## Abstract

The genus *Aletris* L. (Nartheciaceae) encompasses approximately 21–24 species distributed in East Asia and North America, yet taxonomic ambiguity persists due to overlapping morphological traits among closely related species. During fieldwork in southeastern Xizang, China, a morphologically distinct candidate species, *Aletris medogensis*, was discovered. To validate its taxonomic status and explore evolutionary relationships within East Asian *Aletris*, we integrated detailed morphological observation with comparative chloroplast phylogenomics. The newly proposed species is characterized by creeping stolons, narrow leaves, and glandular‐pubescent inflorescences. Comparative analysis of 14 East Asian *Aletris* complete chloroplast genomes revealed a conserved quadripartite structure with species‐specific variations, including pseudogenization of *ycf1*, loss of *rrn4.5*, and shifts in IR boundaries. Phylogenomic analyses strongly supported *A. medogensis* as a distinct species closely related to *A. alpestris* . We identified 18 hypervariable regions as potential molecular markers and detected signals of positive selection in genes *ccsA* , *cemA* , and *rps12*, suggesting adaptive evolution. This study confirms the recognition of *A. medogensis* as a new species endemic to the eastern Himalayas and demonstrates the utility of chloroplast genomics in resolving taxonomic complexity and understanding evolutionary mechanisms in *Aletris*.

## Graphical abstract

Based on morphological and chloroplast phylogenomic analyses, we describe *Aletris medogensis* as a new species endemic to the eastern Himalayas. Comparative genomics revealed structural variations, hypervariable markers, and signatures of positive selection in key genes, providing robust evidence for species delimitation and insights into the evolutionary mechanisms of *Aletris*. This study highlights the power of integrating traditional and molecular approaches in plant systematics.

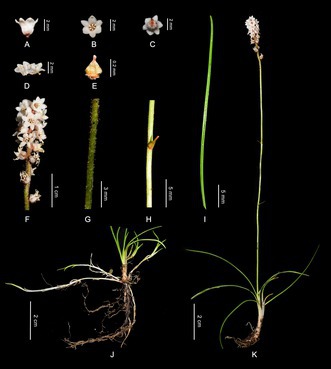

**Abbreviations**
BI: Bayesian inference
cp: chloroplast
DD: data deficient
gDNA: genomic DNA
IR: inverted repeat
LSC: large single copy
LSR: long sequence repeat
ML: maximum likelihood
PCG: protein‐coding gene
PIP: parsimony informative site
SNP: single nucleotide polymorphism site
SSC: small single copy
SSR: simple sequence repeat
SVS: singleton variable site

## 1 Background

The genus *Aletris* L. was established by Linnaeus (1753), with *Aletris farinosa* L. from southeastern North America designated as the type species. It was traditionally placed in the family Liliaceae Juss. (Ambrose 1980; Wu et al. 2003), but molecular phylogenetic studies have since shown closer relationships to the genera *Lophiola*, *Metanarthecium*, *Narthecium*, and *Nietneria*, leading to their reclassification into Nartheciaceae by Caddick et al. (2002). *Aletris* is the largest genus in this family, with approximately 21–24 species found in East Asia and North America (Zhao et al. 2012). In China, 15 species and one variety have been recorded, nine of which are endemic, primarily distributed in the central and southwestern regions.

*Aletris* species typically grow on mountain slopes, roadsides, and shrublands. They prefer moist, shaded environments but can tolerate drought and adapt to various conditions (Yang et al. 2024; Akahori et al. 1971). These perennial herbs possess fibrous roots and basal rosettes of grass‐like leaves (lanceolate to linear). A simple, erect scape bears a terminal raceme of bisexual flowers. Each flower has a bract and a smaller bracteole on the pedicel, a 6‐lobed perianth (white to golden‐orange; cylindrical to campanulate) with rough abaxial surfaces. Six short stamens insert on the perianth tube, and the 3‐lobed stigma yields a beaked, loculicidal capsule enclosed by the persistent perianth (Liang and Turland 2000; Nong et al. 2024). Many *Aletris* species are of ecological and medicinal importance, traditionally used to treat coughs, hemoptysis, dysmenorrhea, and pulmonary abscesses (Li et al. 2014; Challinor et al. 2015).

**FIGURE 1:**
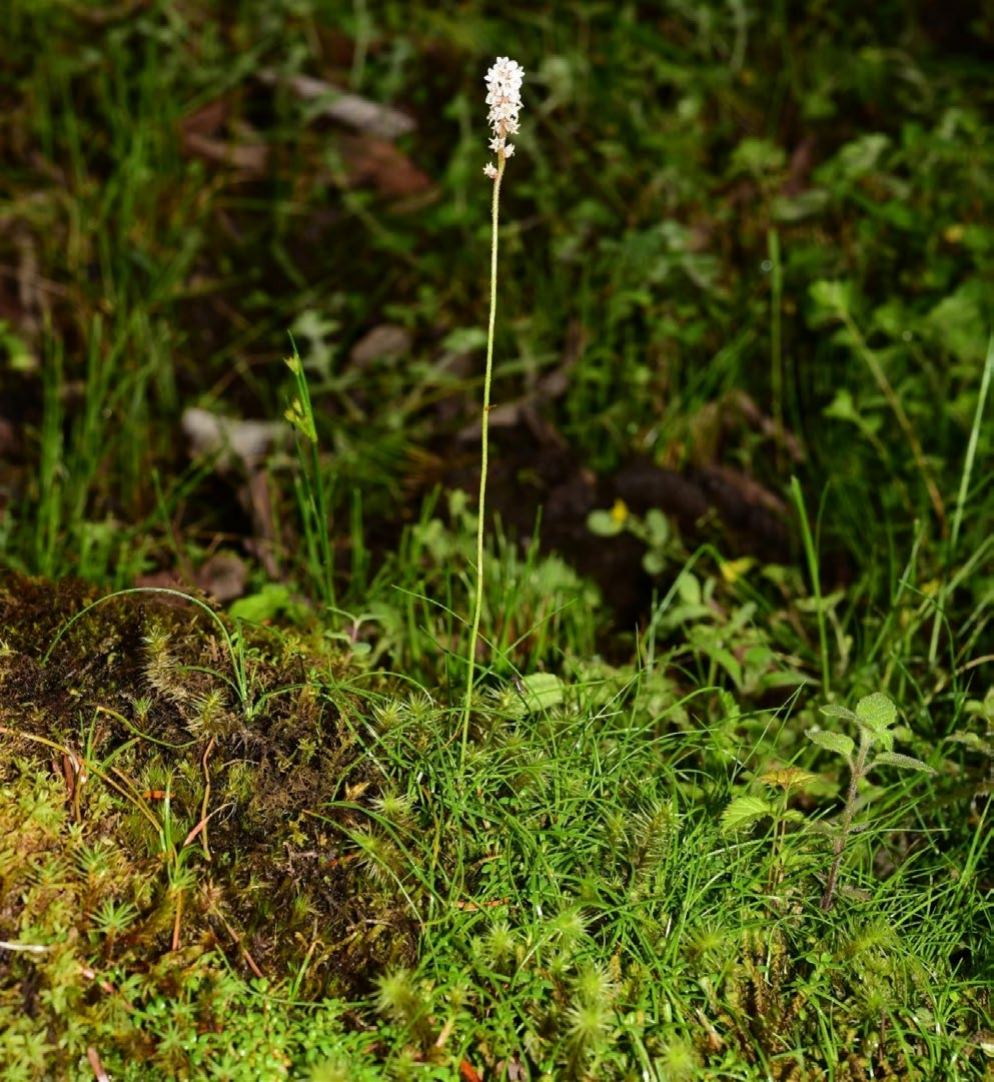
Habit of *Aletris medogensis* on moss‐covered rocks along streams (photographed by Wen‐Bin Ju).

During a 2024 field investigation in southeastern Xizang, we encountered an unusual *Aletris* species growing on moss‐covered rocks along streams (Figure 1). It resembled *A. pauciflora* var. *pauciflora* (Klotzsch) Hand.‐Mazz. (Brotherus and Handel‐Mazzetti 1936), *A. pauciflora* var. *khasiana* F. T. Wang & Tang (Wang and Tang 1978), and *A. alpestris* Diels (Adolf 1905) in having rosette‐like clustered leaves, perianth glandular, with lobes shorter than or equal to the perianth, and the bract and bracteole borne at the apex of the pedicel. However, it differed in its rhizomes horizontally creeping from the base of the plant, narrower linear leaves, inflorescences with glandular hairs, and shorter pedicels, bracts, and bracteoles. We propose it as a new species, *Aletris medogensis* W.B.Ju, Y.L.Qiu & Bo Xu, based on morphological traits and molecular phylogenetic analysis derived from the chloroplast (cp) genome.

In recent years, comparative chloroplast genome analysis has emerged as an essential tool in plant phylogenetics and species identification due to its highly conserved structure, maternal inheritance, and relatively slow evolutionary rate (Zhao et al. 2019; Dopp et al. 2021; Lu et al. 2016). Repeat sequences, inverted repeat (IR) boundaries shift, and nucleotide diversity sites within the chloroplast genome can reveal hypervariable regions and develop molecular markers to evaluate genomic evolutionary history and facilitate species delimitation (Keller et al. 2017; Sabir et al. 2014). Furthermore, the chloroplast genome is not merely a neutral phylogenetic marker; it plays a fundamental role in photosynthesis, stress response, and other essential metabolic pathways. Analyzing selective pressures on chloroplast genes can therefore reveal signatures of adaptive evolution directly linked to environmental factors, such as light intensity, temperature, and water availability, which are particularly relevant for species inhabiting specific niches like the montane ecosystems of the Himalayas (Dong et al. 2018; Rui et al. 2025).

In the genus *Aletris*, although traditional morphological classification has amassed considerable knowledge, taxonomic controversies persist among some closely related species due to overlapping morphological traits and limited molecular data (Zhao et al. 2012; Liang and Turland 2000; Nong et al. 2024). In this study, we first described the morphological traits of *Aletris medogensis* in detail and presented its complete chloroplast (cp) genome for the first time. Then, comparative genomics and phylogenomic analyses were conducted by integrating the previously published cp genome data of East Asian *Aletris* species and related genera in Nartheciaceae, with the following objectives: (1) assess the validity of the newly proposed species status for *Aletris medogensis*; (2) characterize the global structural features and investigate variations in repeat elements among cp genomes; (3) identify highly variable regions suitable for species identification and phylogenetic studies; (4) reconstruct a robust phylogeny of East Asian *Aletris* species and its relatives within Nartheciaceae; and (5) investigate adaptive evolution patterns of cp genes in *Aletris*. This study confirms the distinct taxonomic status of *A. medogensis* , reveals the genetic differentiation of East Asian *Aletris* cp genomes, and sheds light on the evolutionary history and adaptive mechanisms within Nartheciaceae. This integrative framework offers new perspectives for taxonomic studies and biodiversity conservation in complex plant groups.

## 2 Materials and Methods

### 2.1 Sampling and Morphological Comparison

Specimens of *Aletris medogensis* were collected from Hanmi to Xiaoyandong, opposite Beibeng Township, Medog County, Nyingchi City, Xizang, China. The voucher specimen was deposited in the Herbarium of the Chengdu Institute of Biology (CDBI), Chinese Academy of Sciences (https://cib.cas.cn/zzjg/zcbm/bbg/, Bo Xu, xubo@cib.ac.cn), under the accession number CDBI0298322 (Figure S1). Specimens of related species were examined from herbaria PE, HNWP, WUK, SZ, and SM for comparative analysis. Morphological data, including leaves/bract‐like leaves length and width, scape size, inflorescence length, and floral parts (Table 1), were precisely quantified using ImageJ v1.48 (Schneider et al. 2012).

**TABLE 1:** Morphological comparison of *Aletris medogensis* and related taxa.

Character	*A. medogensis*	*A. alpestris*	*A. pauciflora* var. *pauciflora*	*A. pauciflora* var. *khasiana*
Plant	Tillering with horizontally creeping stolons	None	None	None
Leaves	2.5–9 cm × 0.45–0.9 mm, few and slender, linear	1.5–8 cm × 2–4 mm, numerous, densely tufted, linear‐lanceolate	5–25 cm × 2–8 mm, few and laxly tufted, linear‐lanceolate to linear	5–10 cm × 5–7.5 mm, linear‐lanceolate to linear
Scape size	3.5–16 cm × 0.7–1 mm	7–20 cm × 0.5–1 mm	3.5–40 cm × 1.5–2 mm (6.5–8.5 cm × 1.5–2 mm)	7‐15 cm × 2–2.5 mm
Scape hairy	Glabrous, glandular are sparse near the inflorescence	Sparsely puberulent	Densely puberulent	Densely puberulent
Bract–like leaves	0.2–0.6 cm long	Less than 1 cm long	1.5–5.0 cm long	1.5–5.0 cm long
Inflorescence	raceme 1.5–2.5 cm, densely 10–14‐flowered, densely glandular hairs	Raceme 1–4 cm, laxly 4–10‐flowered, rachis puberulent	Raceme 1–5 cm, densely to laxly 4–6‐flowered, rachis pubescent	Raceme 1–5 cm, densely to laxly 18–20‐flowered, rachis pubescent
Bract and bracteole	1.0–2.0 mm long, shorter than flower	1.5–4.0 mm long, shorter than flower	3–20 mm long, one of them 1–4 × flower length	3.5–7.0 mm long, subequaling or slightly exceeding the flower
Pedicel	Less than 0.5 mm long	2–4 mm long	1–12 mm long	1–12 mm long
Perianth	Glabrous, lobes ovate, parted nearly to 1/2 of its length	Glabrous but often densely papillose, lobes lanceolate, parted nearly to 1/2 of its length	Glabrous, lobes oblong‐ovate to lanceolate, parted nearly to 1/4 of its length	Glabrous, lobes oblong‐ovate to lanceolate, parted nearly to 1/4 of its length
Capsule	Apex abruptly narrowed	Apex abruptly narrowed	Apex of valves gradually narrowed	Apex of valves gradually narrowed

### 2.2 Sequencing, Assembly, Annotation, and Comparison

The total genomic DNA (gDNA) was extracted from the dried leaves of *Aletris medogensis* using a modified CTAB method (Porebski et al. 1997). The gDNA was enzymatically fragmented into 200–500 bp fragments, followed by end‐repair, poly‐A tail addition, and adapter ligation. After PCR amplification, a small‐fragment DNA library was constructed and sequenced on the Illumina NovaSeq 6000 platform (Illumina, San Diego, CA, USA) in PE150 mode. To obtain high‐quality sequences, fastp v0.23.2 (Chen et al. 2018) was used to filter out all low‐quality reads, including those with adapters, more than 20% of bases having Phred quality < 5, and reads with > 10% N content. The chloroplast (cp) genome was assembled using GetOrganelle v1.7.7.7.1 (Jin et al. 2020) and annotated using PGA (Qu et al. 2019), with *Aletris pauciflora* var. *pauciflora* as a reference. A Python script (Appendix S1) was used to examine gene length and count, as well as the start and stop codons. In addition, Geneious Prime 8 software (https://www.geneious.com) was employed to determine the pseudogenes. The final circular map was drawn using OGDRAW v1.3.1 (Greiner et al. 2019). In addition, the genomic data of other closely related species within Nartheciaceae, including 13 *Aletris*, 1 *Metanarthecium*, 1 Nartheciaceae, and 1 *Dioscorea* accession (Table 2), were also downloaded and curated. Specifically, genomes with problematic annotations were systematically re‐annotated using our standardized PGA protocol to ensure data uniformity.

**TABLE 2:** Characteristics of 17 Nartheciaceae complete chloroplast genomes, including 1 newly generated accession (*Aletris medogensis*) and the previously published 16 accessions.

Species name	Size (bp)				GC content (%) total (LSC/SSC/IR)	No. of genes (PCGs/tRNA/rRNA)	GenBank accession
	Total	Large single‐copy region (LSC)	Small single‐copy region (SSC)	Inverted repeat (IR)			
*Aletris medogensis*	154,557	83,194	18,167	26,598	37.38 (35.26/31.18/42.82)	131 (85/38/8)	PV472299
*Aletris megalantha*	154,704	83,265	18,113	26,663	37.44 (35.33/31.27/42.82)	131 (85/38/8)	MW080684
*Aletris fauriei* ^a^	154,440	83,501	18,183	26,378	37.5 (35.45/31.31/42.88)	131 (85/38/8)	NC_033412
*Aletris spicata*	154,999	83,511	18,160	26,664	37.48 (35.4/31.27/42.84)	131 (85/38/8)	NC_033411
*Aletris gracilis*	154,201	83,018	17,855	26,664	37.45 (32.44/31.26/42.84)	131 (85/38/8)	PP988376
*Aletris stenoloba*	154,993	83,505	18,160	26,664	37.48 (34.01/31.28/42.84)	131 (85/38/8)	PP988377
*Aletris scopulorum*	154,167	82,706	18,151	26,655	37.49 (32.93/31.22/42.84)	131 (85/38/8)	PP988375
*Aletris pedicellata*	154,204	83,021	17,855	26,664	37.45 (32.44/31.26/42.83)	131 (85/38/8)	PP988374
*Aletris pauciflora* var. *khasiana* ^a^	154,309	83,076	23,519	23,857	37.42 (32.42/33.44/43.11)	130 (85/38/7)	PP988373
*Aletris pauciflora* var. *pauciflora*	154,205	83,022	17,855	26,664	37.45 (32.44/31.26/42.84)	131 (85/38/8)	PP988372
*Aletris laxiflora*	154,468	83,043	18,003	26,711	37.47 (31.31/31.32/42.86)	131 (85/38/8)	PP988370
*Aletris glandulifera*	155,143	83,583	18,248	26,656	37.39 (32.86/31.17/42.83)	131 (85/38/8)	PP988368
*Aletris glabra*	154,201	83,018	17,855	26,664	37.45 (32.44/31.25/42.84)	131 (85/38/8)	PP988367
*Aletris alpestris*	154,551	83,158	18,093	26,650	37.51 (33.69/31.37/42.85)	131 (85/38/8)	PP988366
*Metanarthecium luteoviride*	153,777	82,852	17,777	26,574	37.38 (35.17/31.19/54.77)	130 (84/38/8)	KT895904
*Narthecium ossifragum* ^a^	155,312	84,051	18,135	26,563	37.57 (35.47/31.63/39.59)	130 (85/37/8)	OY986978
*Dioscorea futschauensis*	153,946	83,979	18,909	25,529	37.21 (35.04/31.21/42.99)	124 (86/38/0)	NC_039808

^a^ We re‐annotated the genome based on the sequences from NCBI.

To compare the contraction and expansion of inverted repeat regions among the 14 cp genomes of *Aletris* and its closely related genera, we identified and visualized boundaries of large single copy (LSC), small single copy (SSC), and IRa/IRb regions in the 17 cp genomes using IRscope (Amiryousefi et al. 2018). Furthermore, to explore whether there were additional alterations, such as gene rearrangements or inversions, a synteny analysis of the *Aletris* cp genomes was performed utilizing the mVISTA program (Mayor et al. 2000).

### 2.3 Repeat Sequence Analysis

Simple sequence repeats (SSRs) in *Aletris medogensis* and the 13 published *Aletris* accessions (Table 2) were identified using MISA software (Beier et al. 2017), with threshold settings of 10 repeat units for mononucleotides, 6 for dinucleotides, 5 for trinucleotides, 4 for tetranucleotides, and 3 for penta‐ and hexanucleotides. Then, long sequence repeats (LSRs), including forward (F), reverse (R), palindrome (P), and complementary (C) repeats were detected within the LSC, IRa/b, and SSC regions of the *Aletris* chloroplast genomes using the REPuter program (Kurtz et al. 2001) with a maximum number of computed repeats of 5000 and minimal repeat size of 30 bp.

### 2.4 Molecular Marker Identification

The initial alignment of the 13 *Aletris* complete chloroplast genomes was conducted using MAFFT v7 (Katoh and Standley 2013). Subsequent identification of hypervariable loci with potential phylogenetic utility was detected based on a sliding‐window analysis method to quantify nucleotide diversity (Pi) in *Aletris*, with a window length of 600 bp and a step size of 200 bp. A Python script (Appendix S2) was developed for identifying hypervariable regions. Adjacent windows with a top 5% Pi value and several single nucleotide polymorphism sites (SNPs) > 25 were concatenated into contiguous hypervariable regions. The final characterization of these regions included quantitative assessments of SNPs, singleton variable sites (SVSs), parsimony informative sites (PIPs), and mean Pi values of contiguous hypervariable regions.

### 2.5 Phylogenetic Relationship Reconstruction

The complete chloroplast genome sequences of *A. medogensis* and 16 published species were retrieved from GenBank (https://www.ncbi.nlm.nih.gov/genbank/; Table 2) and aligned with MAFFT. After removing poorly aligned regions using Gblocks v0.91b (Castresana 2000), the best‐fit nucleotide substitution model (TVM+I+G) was determined by jModelTest v2.1.10 (Darriba et al. 2012). Maximum likelihood (ML) and Bayesian inference (BI) trees were then constructed using IQ‐tree v2.1.4 (Nguyen et al. 2015) and MrBayes v3.2.7a (Ronquist et al. 2012), respectively, based on the best‐fit model. *Dioscorea futschauensis* was set as the outgroup. Finally, the resulting trees were visualized and further landscaped in FigTree v1.4.4 (https://github.com/rambaut/figtree/releases/tag/v1.4.4).

### 2.6 Analysis of Selective Pressure

Given the central role of the chloroplast in photosynthesis and its interaction with environmental stressors, signatures of positive selection in its genes can provide insights into molecular adaptations at the intersection of metabolism and ecology. To this end, we calculated the nonsynonymous (*d*_N_) and synonymous (*d*_S_) substitution rates (*ω*) for each PCG. In general, *ω* > 1 indicates that the gene is under positive selection, which may drive species adaptation; *ω* = 1 implies neutral evolution, where no selection is acting; and *ω* < 1 reflects negative or purifying selection, with lower values signifying stronger selective constraints (Wang et al. 2010).

We first retrieved the PCGs shared by 17 Nartheciaceae species via CPStools v2.0.2 (Huang et al. 2024) and translated them into amino acid sequences. Homologous amino acid sequences in 17 cp genomes were then aligned using ParaAT v2.0 (Zhang et al. 2012), and the corresponding CDS alignments (codon‐based) were generated with the Epal2nal.pl script from ParaAT. Finally, KaKs_Calculator v3.0 (Wang et al. 2010) software was employed to calculate *ω* values for all PCGs in each species pair.

## 3 Results

### 3.1 Morphological Analyses

Morphological observations of both fresh samples from the field and dried herbarium specimens revealed that the new species exhibited morphological affinities with *Aletris alpestris*, *A. pauciflora* var. *pauciflora*, and *A. pauciflora* var. *khasiana*. The new species shares several characteristics with *A. alpestris* , including features of the plant, inflorescence structure, involucral bracts, perigone segments, corolla tube, and ovary. It also shows similarities to *A. pauciflora* var. *pauciflora* and *A. pauciflora* var. *khasiana* in leaf shape, stamens, and lobes. However, the new species is morphologically distinct from three taxa, characterized by its narrower leaves, obtuse apex, basal division, the inflorescence axis being densely covered with glandular trichomes, and two bracts that are both shorter than the flowers and unequal in length. Detailed morphological comparisons are provided in Table 1.

### 3.2 Characteristics of the *Aletris* Chloroplast Genomes

This study generated and deposited a new chloroplast genome of *Aletris medogensis* in GenBank (accession no. PV472299), along with 13 published genomes (two of which were re‐annotated) for comparison (Table 2). The results indicated that the whole cp genomes of *Aletris* ranged from 154,167 ( *A. scopulorum* ) to 155,143 bp ( *A. glandulifera* ), exhibiting a typical quadripartite structure consisting of two IR regions (IRa and IRb) of 23,857–26,711 bp, an LSC region of 82,706–83,583 bp, and an SSC region of 17,855–23,519 bp (Table 2). The GC contents of the *Aletris* cp genomes were similar (37.38%–37.51%), with the IRs showing the highest GC levels (42.82%–43.11%), followed by the LSC (31.31%–35.45%) and SSC regions (31.17%–33.44%; Table 2).

**FIGURE 2:**
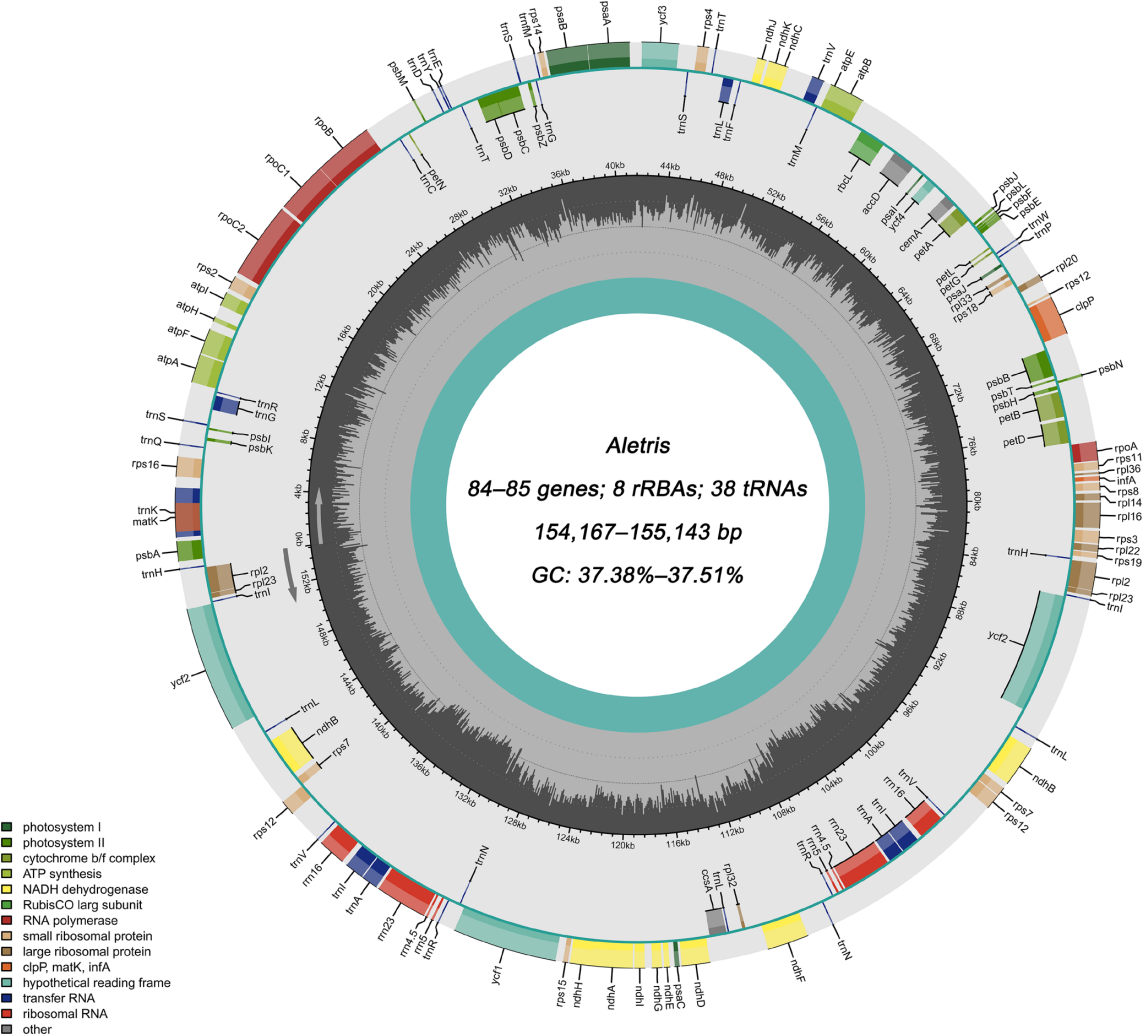
The chloroplast genome map of East Asian *Aletris* species. The outer circle shows distribution of genes (different colors represent different roles). The arrows indicate the transcription directions of the genes inside and outside of the circle. The gray inner circle represents the GC content.

The cp genomes of *Aletris* are highly similar in terms of gene content, with most encoding 131 genes, including 85 protein‐coding genes (PCGs), 38 tRNA genes, and 8 rRNA genes (all located in the IRs; Table 2; Figure 2). Two species had few missing genes and/or pseudogenes (Table 3). Specifically, a *rrn4.5* gene is missing in *Aletris pauciflora* var. *khasiana* and an additional *ycf1* gene is a pseudogene in *A. fauriei* (Table 2). Among the 85 PCGs, 73 were unique, and six (*ndhB*, *rpl2*, *rpl23*, *rps7*, *rps12*, and *ycf2*) were duplicated due to their location in the IRs. Likewise, 22 of the tRNA genes are unique, while eight tRNA genes (*trnA‐UGC*, *trnH‐GUG*, *trnI‐CAU*, *trnI‐GAU*, *trnL‐CAA*, *trnN‐GUU*, *trnR‐ACG*, and *trnV‐GAC*) and all four rRNA genes (rrn23, rrn16, rrn5, and rrn4.5) were duplicated. Additionally, a total of 18 genes were found to contain introns across the 14 cp genomes, with nine PCGs (*atpF*, *ndhA*, *ndhB*, *petB*, *petD*, *rpl2*, *rpl16*, *rpoC1*, and *rps16*) and six tRNA genes (*trnA‐UGC*, *trnG‐UCC*, *trnI‐GAU*, *trnK‐UUU*, *trnL‐UAA*, *trnV‐UAC*) containing one intron, while only three PCGs (*rps12*, *ycf3*, and *clpP*) contained two introns (Table 3).

**TABLE 3:** Summary of gene contents present in the *Aletris* chloroplast genomes.

Category	Gene group	Gene name
Photosynthesis	Subunits of photosystem I	*psaA*, *psaB*, *psaC*, *psaI*, *psaJ*
	Subunits of photosystem II	*psbA*, *psbB*, *psbC*, *psbD*, *psbE*, *psbF*, *psbH*, *psbI*, *psbJ*, *psbK*, *psbL*, *psbM*, *psbN*, *psbT*, *psbZ*
	Subunits of NADH dehydrogenase	*ndhA* (1), *ndhB* (1) (×2), *ndhC*, *ndhD*, *ndhE*, *ndhF*, *ndhG*, *ndhH*, *ndhI*, *ndhJ*, *ndhK*
	Subunits of cytochrome *b*/*f* complex	*petA*, *petB* (1), *petD* (1), *petG*, *petL*, *petN*
	Subunits of ATP synthase	*atpA*, *atpB*, *atpE*, *atpF* (1), *atpH*, *atpI*
	Large subunit of rubisco	*rbcL*
Self‐replication	Proteins of large ribosomal subunit	*rpl14*, *rpl16* (1), *rpl2* (1) (×2), *rpl20*, *rpl22*, *rpl23* (×2), *rpl32*, *rpl33*, *rpl36*
	Proteins of small ribosomal subunit	*rps11*, *rps12* (2) (×2), *rps14*, *rps15*, *rps16* (1), *rps18*, *rps19*, *rps2*, *rps3*, *rps4*, *rps7* (×2), *rps8*
	Subunits of RNA polymerase	*rpoA*, *rpoB*, *rpoC1* (1), *rpoC2*
	Ribosomal RNAs	*rrn16* (×2), *rrn23* (×2), *rrn5* (×2), *rrn4.5* (×2)^a^
	Transfer RNAs	*trnA‐UGC* (1) (×2), *trnC‐GCA*, *trnD‐GUC*, *trnE‐UUC*, *trnF‐GAA*, *trnG‐GCC*, *trnG‐UCC* (1), *trnH‐GUG* (×2), *trnI‐CAU* (×2), *trnI‐GAU* (×2), *trnK‐UUU* (1), *trnL‐CAA* (×2), *trnL‐UAA* (1), *trnL‐UAG*, *trnM‐CAU*, *trnN‐GUU* (×2), *trnP‐UGG*, *trnQ‐UUG*, *trnR‐ACG* (×2), *trnR‐UCU*, *trnS‐GCU*, *trnS‐GGA*, *trnS‐UGA*, *trnT‐GGU*, *trnT‐UGU*, *trnV‐GAC* (×2), *trnV‐UAC* (1), *trnW‐CCA*, *trnY‐GUA*, *trnfM‐CAU*
Other genes	Maturase	*matK*
	Protease	*clpP* (2)
	Envelope membrane protein	*cemA*
	Acetyl‐CoA carboxylase	*accD*
	c‐type cytochrome synthesis gene	*ccsA*
	Translation initiation factor	*infA*
	Conserved hypothetical chloroplast reading frames	*ycf1* ^b^, *ycf2* (×2), *ycf3* (2), *ycf4*

*Note:* (1) Genes with one intron; (2) genes with two introns; (×2) genes with two copies.

^a^ One of the *rrn4.5* genes is missing in *Aletris pauciflora* var. *khasiana*.

^b^ An additional *ycf1* pseudogene in *Aletris fauriei*.

### 3.3 Codon Usage of *Aletris* cp Genomes

**FIGURE 3:**
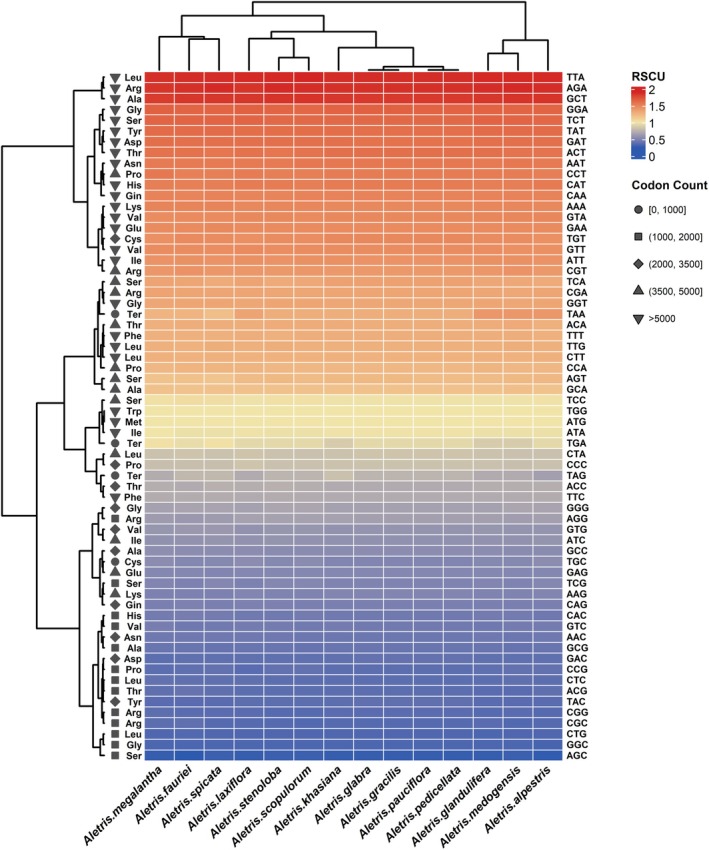
Heatmap of RSCU values among *Aletris* species. The color gradient from blue to red represents the range of RSCU values and different graphics on the left side represent different quantity ranges.

The RSCU analysis revealed a similar codon usage pattern among the 14 *Aletris* species, with each containing all 64 codons that encode 20 amino acids (Figure 3; Table S1). In these genomes, leucine exhibited the highest number (29,369) of codons, while cysteine was the least abundant, with 3288 codons (Figure 3; Table S2). The highest mean RSCU value was identified as UUA (1.88), and the lowest was AGC (mean RSCU = 0.28), which encode leucine and serine, respectively (Figure 3; Table S2). Furthermore, 31 codons were found with a mean RSCU of > 1, of which 29 were A/U‐ending codons; 33 codons were found with a mean RSCU of ≤ 1, of which 30 were G/C‐ending codons. This suggests strong codon usage bias favoring A/U‐ending codons over G/C‐ending codons in *Aletris* chloroplast genomes (Figure 3; Table S2).

### 3.4 SSRs and Long Repeat Sequences of *Aletris* Cp Genomes

**FIGURE 4:**
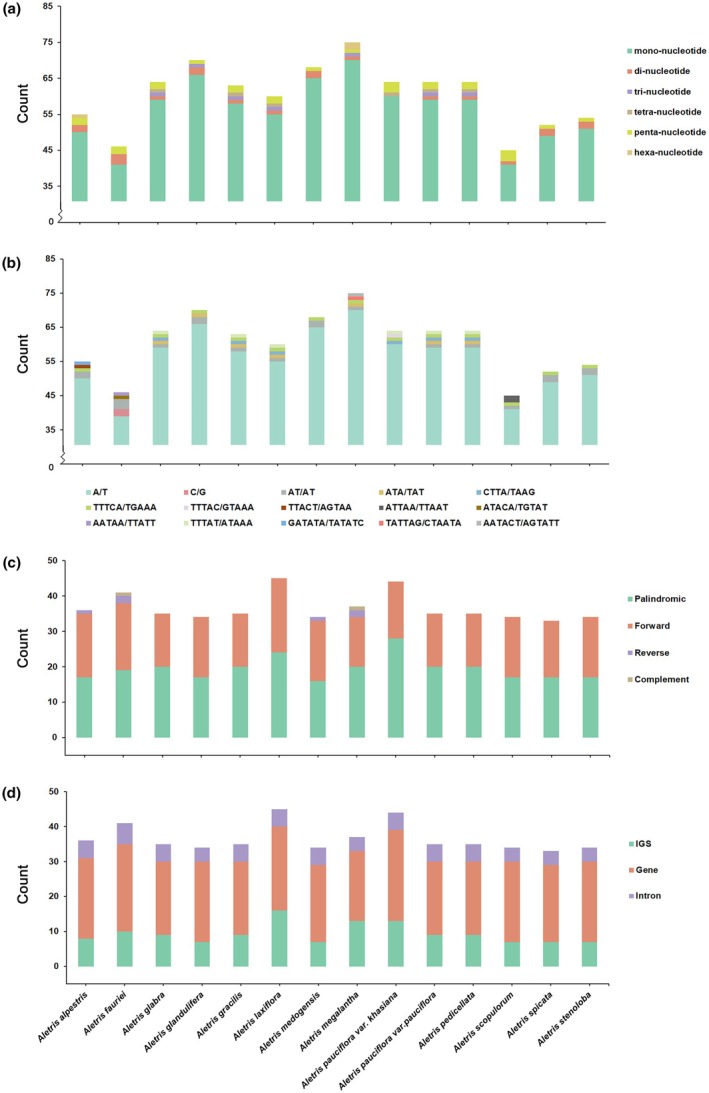
Patterns of simple sequence repeats [SSRs (a, b)] and long sequence repeats [LSRs (c, d)] for the 14 *Aletris* chloroplast genomes. (a) Number of motifs and their abundance of SSRs in each species. (b) Type of motifs and their abundance of SSRs in each species. (c) Type and abundance of LSRs in each species. (d) Number of LSRs at different locations in each genome.

The number of SSRs in the 14 *Aletris* species varied from 45 in *A. scopulorum* to 75 in *A. megalantha*, in which mono‐nucleotide SSRs were the most abundant, followed by tetra‐ and di‐nucleotide SSRs (Figure 4a; Table S3). Among the motifs in the SSRs, A/T were the most frequently occurring motifs, followed by AT/AT and TTTCA/TGAAA motifs (Figure 4b; Table S4). In addition, most of the SSRs were located in the LSC (35–59) and SSC (6–12) regions, and very few were located in the IRa/IRb (1/1–4/4; Table S3). REPuter identified 34–45 repeat sequences with a length > 30 bp, of which palindromic (17–28) and forward (14–21) repeat sequences were most abundant, while complement (0–2) and reverse (0–1) repeat sequences were very rare and almost nonexistent in many species (Figure 4c; Table S5). Most of the repeat sequences (21–26) were located in gene regions of the 14 cp genomes, followed by intergenic regions (7–16, IGS) and introns (4–6; Figure 4d; Table S5). In addition, most of the repeat sequences were less than 50 bp, and a few were larger than 100 bp (Table S6). Notably, *A. pauciflora* var. *khasiana* had a palindrome repeat sequence with a length of 2820 bp (Table S6).

### 3.5 Variance Analysis of the IR Boundaries

**FIGURE 5:**
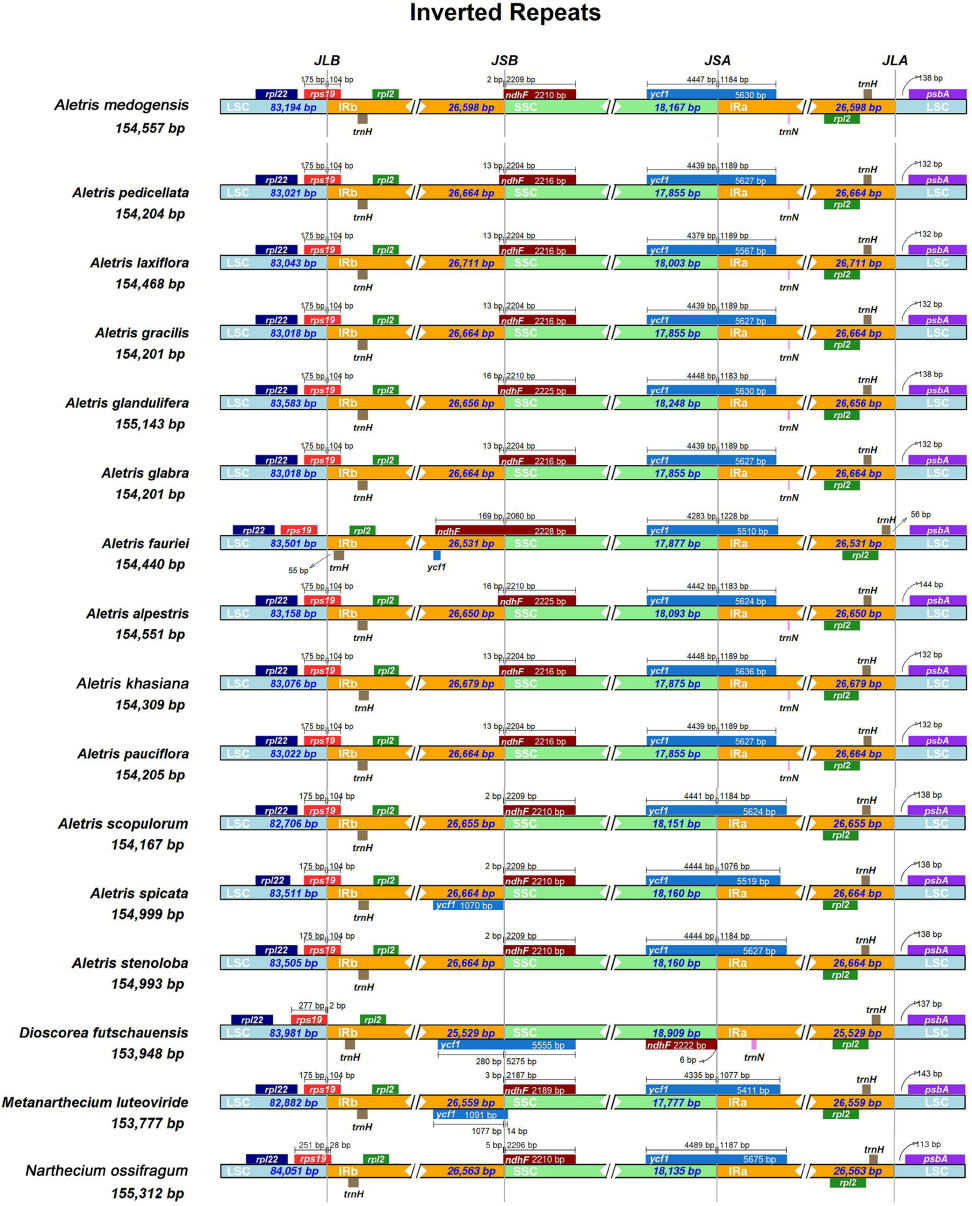
Comparison of LSC, SSC, and IR region boundaries in 17 Nartheciaceae chloroplast genomes.

We compared the IR boundaries among 17 Nartheciaceae cp genomes, including *Dioscorea futschauensis*, *Metanarthecium luteoviride*, *Narthecium ossifragum*, and the 14 *Aletris* accessions mentioned above (Table 2), observing minor variations in gene content, structure, and order at the quadripartite borders (Figure 5; Figure S2). The JLA (IRa‐LSC) and JSA (IRa‐SSC) boundaries were highly conserved in all 14 *Aletris* cp genomes, with the former positioned between *psbA* and *trnH*, and the latter cut through the *ycf1* gene. The distance from the JLA boundary to *psbA* was consistently 138 bp, whereas that to *trnH* varied from 56 to 249 bp (Figure 5). The JLB (IRb‐LSC) boundaries cut through *rps19* in most *Aletris* species, with 104 bp of *rps19* extending into the IRb, while the JLA boundary of *A. fauriei* lay 77 bp away from *rps19* due to the contraction of IRb. Similarly, the *ndhF* gene extended 2–16 bp into the JSB (IRb‐SSC) boundaries in most *Aletris* species except *A. fauriei*, where *ndhF* extended 153 bp into IRb because of IR expansion (Figure 5). Differences in the expansion/contraction of the IRs were also found in other Nartheciaceae species; only 2 and 28 bp of *rps19* extended into the IRb boundary in *Dioscorea futschauensis* and *Narthecium ossifragum*, respectively. In addition, an inversion event involving the *ndhF* and *ycf1* genes in the *Dioscorea futschauensis* cp genome occurred, with the former now extending 6 bp into the JSA, and the latter was cut through by the JSB boundary.

### 3.6 Candidate Molecular Markers Identification

**FIGURE 6:**
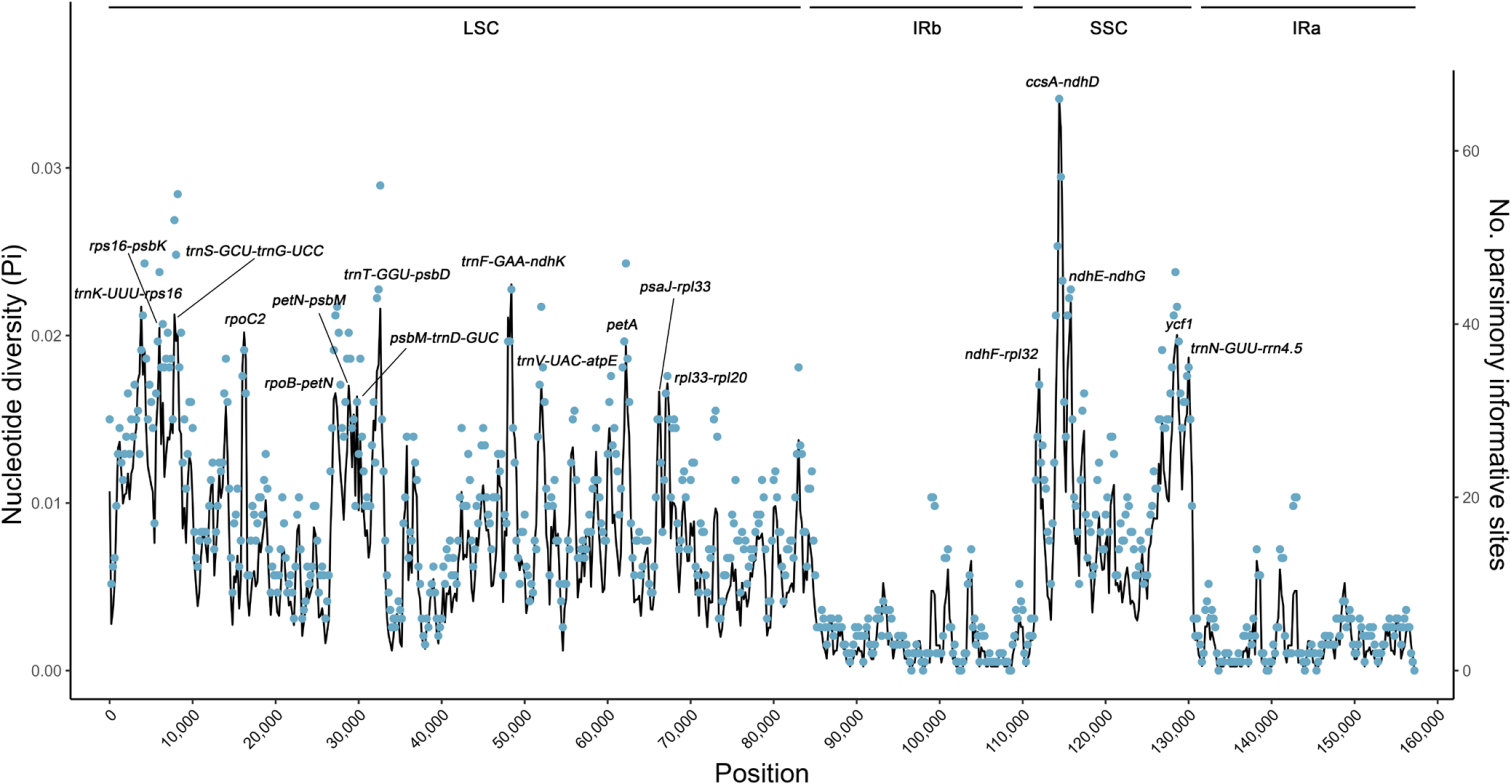
Nucleotide diversity (Pi, black line, vertical left axis) and number of single nucleotide polymorphism sites (blue dots, vertical right axis) of 14 *Aletris* cp genomes based on sliding window analysis. The window length is 600 bp and the step size is 200 bp. The horizontal axis indicates the position of the midpoint of a window. The 18 regions with high diversity (top 5%) are indicated above the peaks.

Using sliding window analysis, 18 hypervariable regions were identified as potential East Asia‐specific molecular markers for *Aletris* taxonomy, primarily occurring in the LSC and SSC regions and ranging from 523 to 2133 bp (Figure 6; Table 4). Among them, 11 intergenic spacers and 2 gene regions are located in the LSC region, 2 intergenic spacers and 1 gene are located in the SSC region, and the remaining intergenic spacer of *trnN‐GUU* and *rrn4.5* (*trnN‐GUU‐rrn4.5*) is positioned in the IRa region (Figure 6). Notably, the *ccsA‐ndhD* intergenic spacer was the longest (2133 bp) and contained the greatest number of parsimony informative sites (PIP, 49) as well as the highest Pi value (0.020851; Table 4). In addition, the *ycf1*, *petA*, and *rpoC2* genes also displayed sufficient variability and could be utilized as molecular markers for species identification and genetic studies within *Aletris* (Figure 6).

**TABLE 4:** Hypervariable regions identified among the 14 cp genomes of *Aletris*.

Start	End	Length	# SNP	# SVS	# PIP	Pi	Gene name
3400	4745	1346	78	50	22	0.016959	*trnK‐UUU‐rps16*
5800	6578	779	48	32	14	0.016218	*rps16‐psbK*
7800	8759	960	78	56	18	0.020378	*trnS‐GCU‐trnG‐UCC*
16,000	16,993	994	42	23	13	0.012838	*rpoC2*
27,000	27,789	790	54	37	14	0.01674	*rpoB‐petN*
28,800	29,361	562	38	26	12	0.017218	*petN‐psbM*
29,800	30,389	590	31	11	20	0.016653	*psbM‐trnD‐GUC*
32,200	33,185	986	64	45	13	0.016593	*trnT‐GGU‐psbD*
48,000	48,892	893	51	32	18	0.016814	*trnF‐GAA‐ndhK*
52,000	52,586	587	42	28	12	0.017529	*trnV‐UAC‐atpE*
62,200	62,744	545	39	30	8	0.015932	*petA*
66,200	66,722	523	26	14	11	0.016847	*psaJ‐rpl33*
67,000	67,779	780	39	23	14	0.01477	*rpl33‐rpl20*
112,000	112,596	597	33	15	17	0.018093	*ndhF‐rpl32*
114,200	116,332	2133	154	104	49	0.020851	*ccsA‐ndhD*
117,400	117,996	597	32	18	14	0.014381	*ndhE‐ndhG*
128,200	129,199	1000	68	42	26	0.018297	*ycf1*
130,000	130,599	600	35	17	18	0.018681	*trnN‐GUU‐rrn4.5*

*Note:* The start and end positions are referred to the alignment sequences of 14 *Aletris* species.

Abbreviations: # PIP, number of parsimony informative sites; # SNP, number of single nucleotide polymorphism sites; # SVS, number of singleton variable sites.

### 3.7 Phylogenetic Relationship of *Aletris*

**FIGURE 7:**
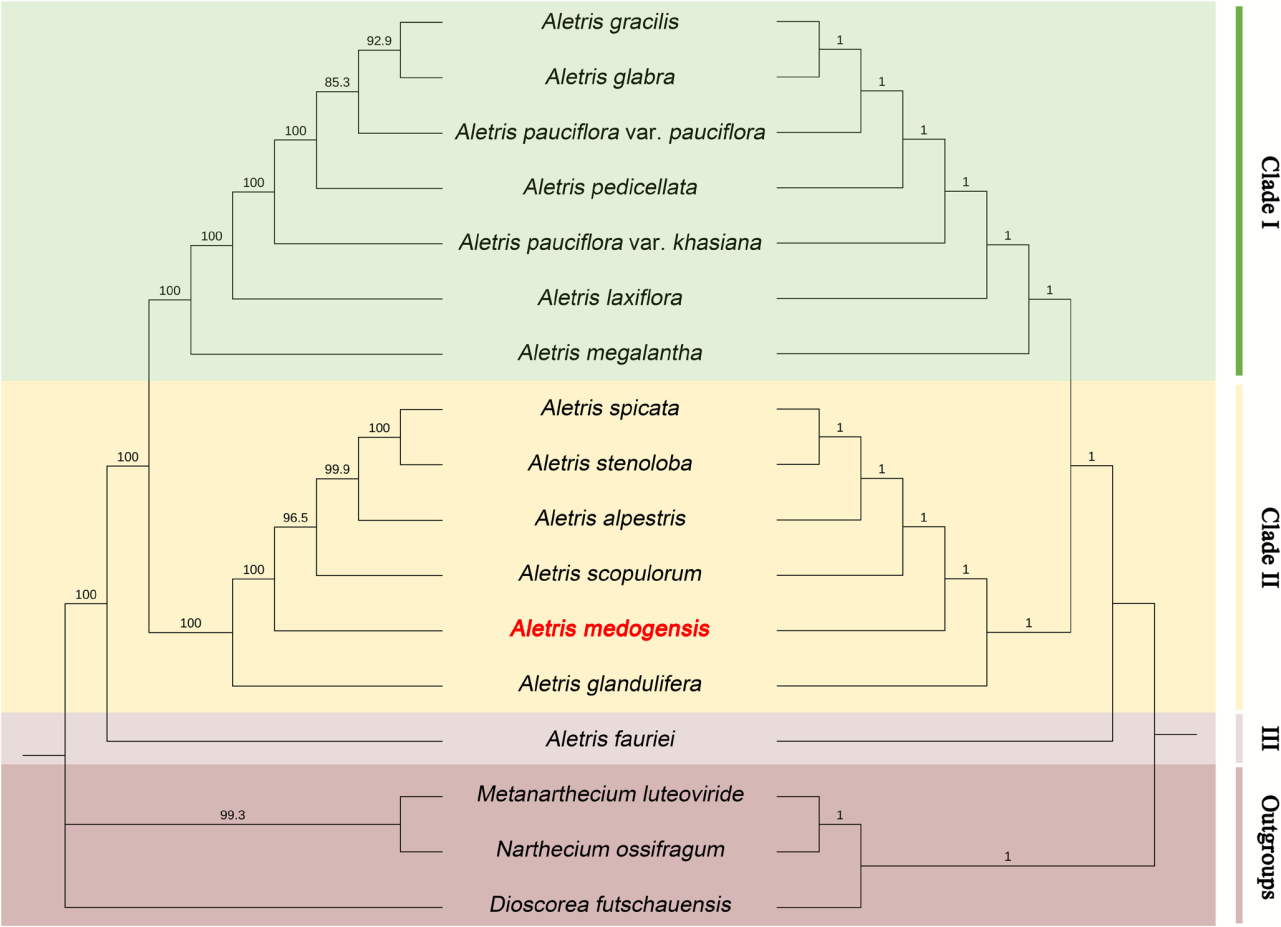
Phylogenetic tree inferred from Maximum Likelihood (ML, on the left) and Bayesian Inference (BI, on the right) methods for East Asian *Aletris* species and their closely related species based on whole chloroplast genomes. The numbers above branches indicate ML bootstrap support (BS) and Bayesian posterior probabilities (PP) respectively.

We found that the relationships among all 14 East Asian *Aletris* species were completely congruent between ML and BI analyses, forming a strongly supported monophyletic group (BS = 100%, PP = 1.0) that was sister to the outgroup taxa: *Dioscorea futschauensis*, *Metanarthecium luteoviride*, and *Narthecium ossifragum* (BS = 100%, PP = 1.0; Figure 7). While the BI analysis fully resolved the outgroup relationships, with *Metanarthecium luteoviride* and *Narthecium ossifragum* forming a sister group that was then sister to *Dioscorea futschauensis* (PP = 1.0), the ML analysis left them as an unresolved polytomy (Figure 7).

Within *Aletris* species, three evolutionary clades emerged with strong support (BS = 100%, PP = 1): *A. fauriei* occupies an isolated position as the earliest‐diverging lineage (Clade III), sister to the remaining species, which can be further subdivided into two additional clades (Figure 7). The new species, *A. medogensis* , clustered as a sister group to a subclade consisting of *A. spicata* , *A. stenoloba*, *A. alpestris* , and *A. scopulorum* , and together as a sister to *A. glandulifera* , forming Clade II. Clade I united *A. gracilis* , *A. glabra* , *A. pauciflora* , *A. pedicellata* , *A. pauciflora* var. *khasiana*, *A. laxiflora* , and *A. megalantha*. Critically, *A. medogensis* exhibited stronger phylogenetic affinity to *A. alpestris* in Clade II than to its morphological relatives *A. pauciflora* var. *pauciflora* and *A. pauciflora* var. *khasiana* in Clade I (Figure 7), revealing significant morphology–molecular discordance.

### 3.8 Selective Pressure of cp Genes in Nartheciaceae

**FIGURE 8:**
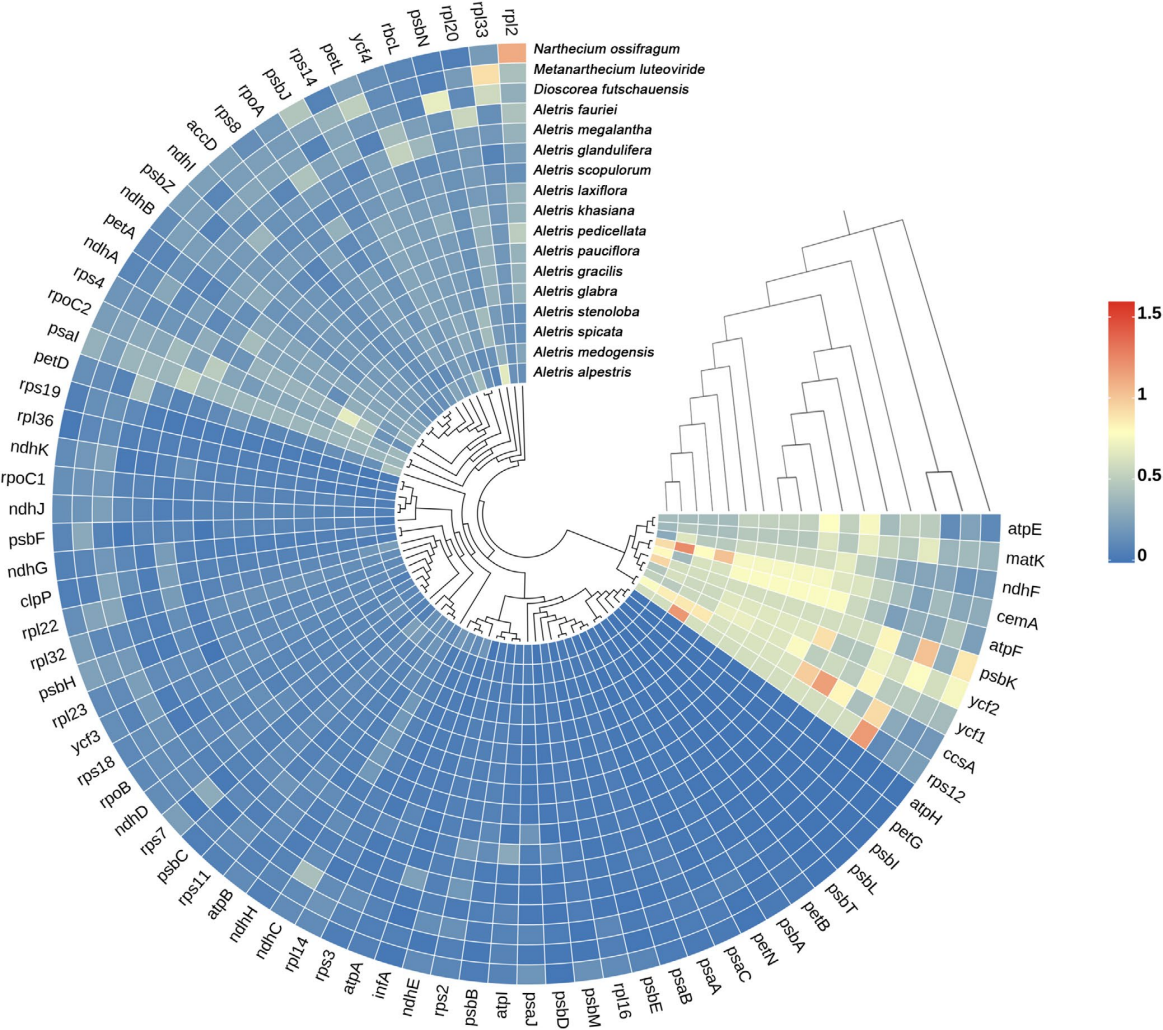
Heatmap of the mean *K*_a_/*K*_s_ values of 77 PCGs shared by 17 Nartheciaceae species.

A total of 77 PCGs shared among 17 Nartheciaceae cp genomes were analyzed for selective pressure, and most of them were subjected to purifying selection (*K*_a_/*K*_s_ < 1; Figure 8; Table S7). By calculating the *K*_a_/*K*_s_ ratios for homologous genes within species pairs, only *ccsA* in *Aletris scopulorum*, *cemA* in *A. medogensis* and *A. stenoloba*, *rps12* in *A. spicata* and *A. fauriei*, *psbK* in *Dioscorea futschauensis*, and *rpl2* in *Narthecium ossifragum* showed signatures of positive selection (*K*_a_/*K*_s_ > 1). These findings suggest that some mutations were positively favored by selection in specific species. Furthermore, eight genes (*atpE*, *matK*, *ndhF*, *cemA*, *atpF*, *ccsA*, *rps12*, and *ycf1*) evolved faster in most *Aletris* species than in the other three Nartheciaceae species branches (Figure 8; Table S7), potentially reflecting species‐specific adaptations.

## 4 Discussion

The discovery of *Aletris medogensis* as a new species (Figure 1), supported by both morphological distinctiveness and chloroplast genome analysis, highlights the importance of integrating traditional taxonomy with modern molecular tools. This study not only expands known endemism in the eastern Himalayas but also provides critical insights into the evolutionary dynamics and genomic features of Nartheciaceae.

### 4.1 Chloroplast Genome Evolution in *Aletris*

Consistent with other angiosperms (Sugiura 1992; Daniell et al. 2016), the comparative analysis of the 14 East Asian *Aletris* cp genomes revealed a conserved quadripartite structure with minor variations in size (154,167–155,143 bp) and gene content (Table 2). Various elements influencing chloroplast structure and function, such as the contraction and expansion of the IR regions, gene insertions, deletions, duplications, inversions, and alterations in introns, have been reported in many species (Tsudzuki et al. 1992; Lin et al. 2012; Li et al. 2020). Notably, the pseudogenization of the *ycf1* gene in *A. fauriei* and the loss of *rrn4.5* in *A. pauciflora* var. *khasiana* are of functional interest. The former is significant given its role in plastid protein import and stress responses, while the latter might reduce ribosomal redundancy in stable riparian niches, potentially enhancing DNA repair capacity in high‐UV montane habitats (Dong et al. 2015; Wei et al. 2024). Additionally, the IR boundary dynamics also observed here—particularly the expansion of *ndhF* into IRb and the contraction of *rps19* from IRb in *A. fauriei* (Figure 5)—reflect species‐specific genomic rearrangements. Such IR contractions/expansions can alter gene dosage and disrupt operon integrity, potentially affecting photosynthetic efficiency (Kim and Lee 2005; Zoschke and Bock 2018).

The strong codon usage bias favoring A/U‐ending codons (Figure 3) aligns with angiosperm‐wide trends but shows intensified bias in Himalayan species (Liu and Xue 2005; Smarda et al. 2014). This likely optimizes translational efficiency under low‐temperature stress, as A/U‐rich transcripts fold less stably, facilitating ribosome binding in cold environments (Chiba et al. 2013; Krasileva et al. 2017). Additionally, the excess of SSRs and LSRs in intergenic regions (Figure 4d) suggests their role in regulating genome plasticity; the 2820 bp palindromic repeat in *A. pauciflora* var. *khasiana* (Table S6) could promote recombination‐driven innovation, potentially accelerating adaptation to microhabitat heterogeneity (Wicker et al. 2007; Oliver et al. 2013).

### 4.2 Phylogenetic Implications for *Aletris* Taxonomy

Although *A. medogensis* possesses a unique combination of morphological characters not found in its morphologically similar relatives (e.g., *A. alpestris* , *A. pauciflora* ), morphological characteristics alone may be insufficient to resolve species boundaries, where subtle differences in floral structures and vegetative traits often overlap among taxa (Table 1). Our chloroplast phylogenomic analysis resolves long‐standing controversies in East Asian *Aletris* systematics. First, *Metanarthecium luteoviride* formed a clade with *Narthecium ossifragum* (PP = 1, Figure 7), providing robust genomic evidence to exclude it from *Aletris*—a conclusion consistent with Zhao et al. (2012) but contradicting fragment‐based studies (Merckx et al. 2008; Fuse et al. 2012). Second, our data confirm that *A. pauciflora* var. *khasiana* and *A. pauciflora* var. *pauciflora* are phylogenetically distinct (Figure 7), validating Zhao et al.'s (2012) proposal to recognize the former as a separate species. Critically, the whole cp genome tree provided robust molecular evidence to corroborate the distinctiveness of *Aletris medogensis* by demonstrating that it was phylogenetically distant from the two varieties of *A. pauciflora* (which were nested within Clade I), and that, despite belonging to the same major clade (Clade II), it was not closely related to *A. alpestris* , which formed a separate subclade (Figure 7). This discordance between morphological affinity and phylogenetic placement underscores the necessity of molecular data to clarify evolutionary relationships in taxonomically challenging groups (Redwan et al. 2015).

While our integrative evidence from morphology and chloroplast phylogenomics robustly supports the recognition of *A. medogensis* as a distinct species, phylogenetic inferences based solely on plastid data may not always reflect the true species tree because of evolutionary processes such as incomplete lineage sorting and introgression, due to its uniparental inheritance. Therefore, the employment of nuclear markers, or a combination of nuclear and plastid data, is strongly advocated in future studies to further validate and refine the phylogenetic relationships proposed here.

### 4.3 Hypervariable Regions and Molecular Markers

The hypervariable regions identified here (Figure 6; Table 4), including *ycf1* (Pi = 0.0183), *petA* (Pi = 0.0183), and *rpoC2* (Pi = 0.0183) gene regions, offer superior resolution for species delimitation compared to traditional markers (e.g., *matK*/*trnL‐F*) (Zhao et al. 2012). For instance, the *A. spicata* –*A. stenoloba* complex could not be resolved in previous studies due to identical sequences (Zhao et al. 2012), whereas our SNPs of the *ycf1* gene clearly distinguish them, implying the plastid gene *ycf1* could serve as a key barcode for *Aletris*. In addition, these hypervariable regions will serve as valuable molecular markers for future phylogenetic and population genetic studies (Wei et al. 2024). The prevalence of SSRs (45–75 per genome) and LSRs (34–45 per genome) underscores the potential role of repetitive elements in shaping cp genome evolution (Tables S3–S5) (Xu et al. 2023). The exceptional 2820 bp palindromic repeat in *A. pauciflora* var. *khasiana* (Table S6) may serve as a hotspot for recombination, contributing to genomic plasticity (Feng et al. 2022).

### 4.4 Selective Pressures and Adaptive Evolution

Positively selected genes are pivotal drivers of evolutionary innovation, enabling organisms to exploit new ecological niches and respond dynamically to changing environments (Moseley et al. 2018). Unlike negative (purifying) selection, which maintains genomic stability by removing deleterious mutations across long evolutionary timescales (Cvijovic et al. 2018), positive selection favors advantageous alleles that confer improved fitness—whether through enhanced metabolic efficiency, novel biochemical pathways, or refined sensory capabilities (Messer and Petrov 2013; Li et al. 2025).

Most protein‐coding genes in *Aletris* are under purifying selection (*ω* < 1; Figure 8; Table S7), consistent with the functional constraints of essential photosynthetic and ribosomal genes. However, positive selection (*ω* > 1) was detected in *ccsA* ( *A. scopulorum* ), *cemA* ( *A. medogensis* and *A. stenoloba*), and *rps12* ( *A. spicata* and *A. fauriei*). These genes, involved in cytochrome synthesis, chloroplast envelope stability, and ribosomal function (Xie and Merchant 1996; Ogawa et al. 1994; Ramundo et al. 2013), have also been found to undergo positive selection in other taxa (e.g., *Ficus*, *Populus*) (Zhang et al. 2022; Shi et al. 2025). Their accelerated evolution might reflect species‐specific adaptations to environmental stressors like high‐altitude habitats or pathogen resistance (Zhang et al. 2020). The faster evolutionary rates of *matK* and *ndhF* in *Aletris* than in other Nartheciaceae genera further highlight lineage‐specific selective regimes, possibly linked to ecological diversification (Barthet and Hilu 2007; Zhao et al. 2017).

### 4.5 Conservation Implications

Despite its restricted distribution in Medog County, *A. medogensis* is currently classified as data deficient (DD) due to insufficient population data. The species' reliance on moss‐covered riparian habitats makes it vulnerable to climate change, which can manifest as altered stream flow regimes and the desiccation of its required microclimate, and to direct anthropogenic disturbances such as tourism development and infrastructure construction along its fragile streamside habitat (Xu et al. 2009; Zhang and Yan 2023). In‐depth surveys are needed across similar ecosystems in the Eastern Himalayas to assess the true distribution and conservation status of this endemic lineage, allowing for essential morphometric comparisons across populations to validate a suite of stable, diagnostic traits. Additionally, the genomic resources generated here, including hypervariable markers and cp genome annotations, will facilitate monitoring efforts and inform conservation strategies for this endemic lineage.

## 5 Conclusions

The integration of morphology, chloroplast genomics, and phylogenomics effectively resolves taxonomic conflicts in *Aletris* and reveals evolutionary mechanisms underlying species diversification. This work enhances our understanding of Himalayan plant diversity and provides a framework for future studies on cryptic species in evolving ecosystems.

## 6 Taxonomic Treatment

***Aletris medogensis*** W.B.Ju, Y.L.Qiu & Bo Xu, sp. nov.

### 6.1 Type

CHINA. Xizang: Motuo County, Beibeng Xiang, from Hanmi to Xiaoyandong on the opposite side, ca. 2931 m, 29°23′25.24′′, 95°06′00.75′′, occurring on moss‐covered rocks along riparian zones. June 9, 2024, Ju Wen‐Bin, Li Jiang‐Tao, Zeng Li‐Ting, and Deng Hui‐Wen YLZB12018 (holotype: CDBI0298322; Figure S1).

### 6.2 Diagnosis

*Aletris medogensis* is morphologically most similar to *Aletris alpestris*, but it can be distinguished from the latter in having narrower leaves (0.45–0.9 mm wide vs. 1–2.5 mm wide) and an obtuse apex (vs. an acuminate apex); the flower pedicels are extremely short, the rachis has densely glandular hairs (vs. sparsely puberulent), the two bracts are unequal in length and shorter than the flowers (vs. one of them 1–4 × flower length). Lobes are obtuse to rounded at the apex (vs. the apex being obtuse to acute).

### 6.3 Description

**FIGURE 9:**
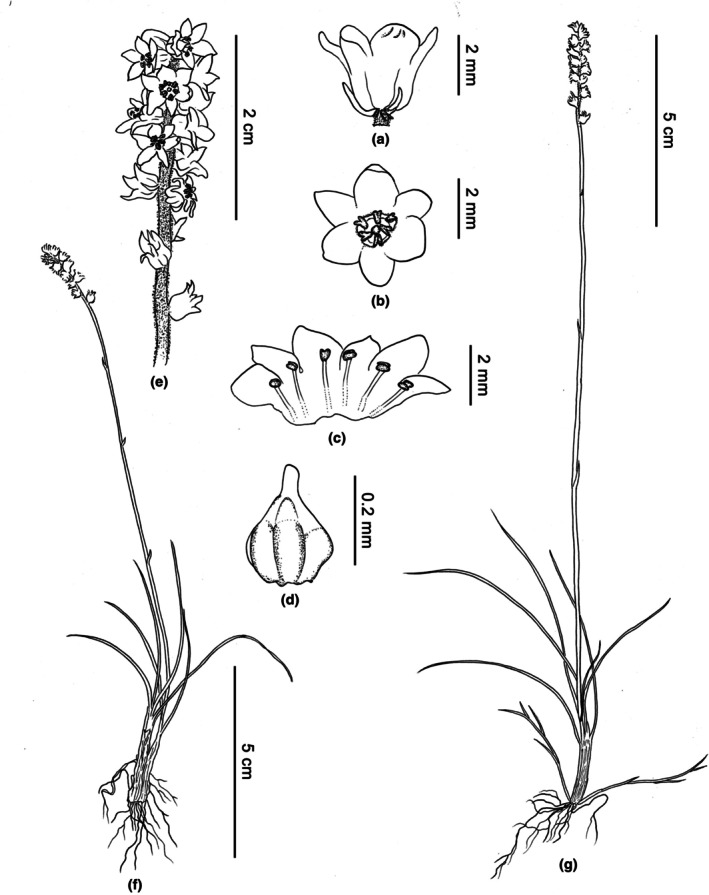
Line drawing of *Aletris medogensis*. Lateral (a) and front (b) views of the flower. (c) Stamens. (d) Ovary and stigma. (e) Inflorescence. (f, g) Plants.

**FIGURE 10:**
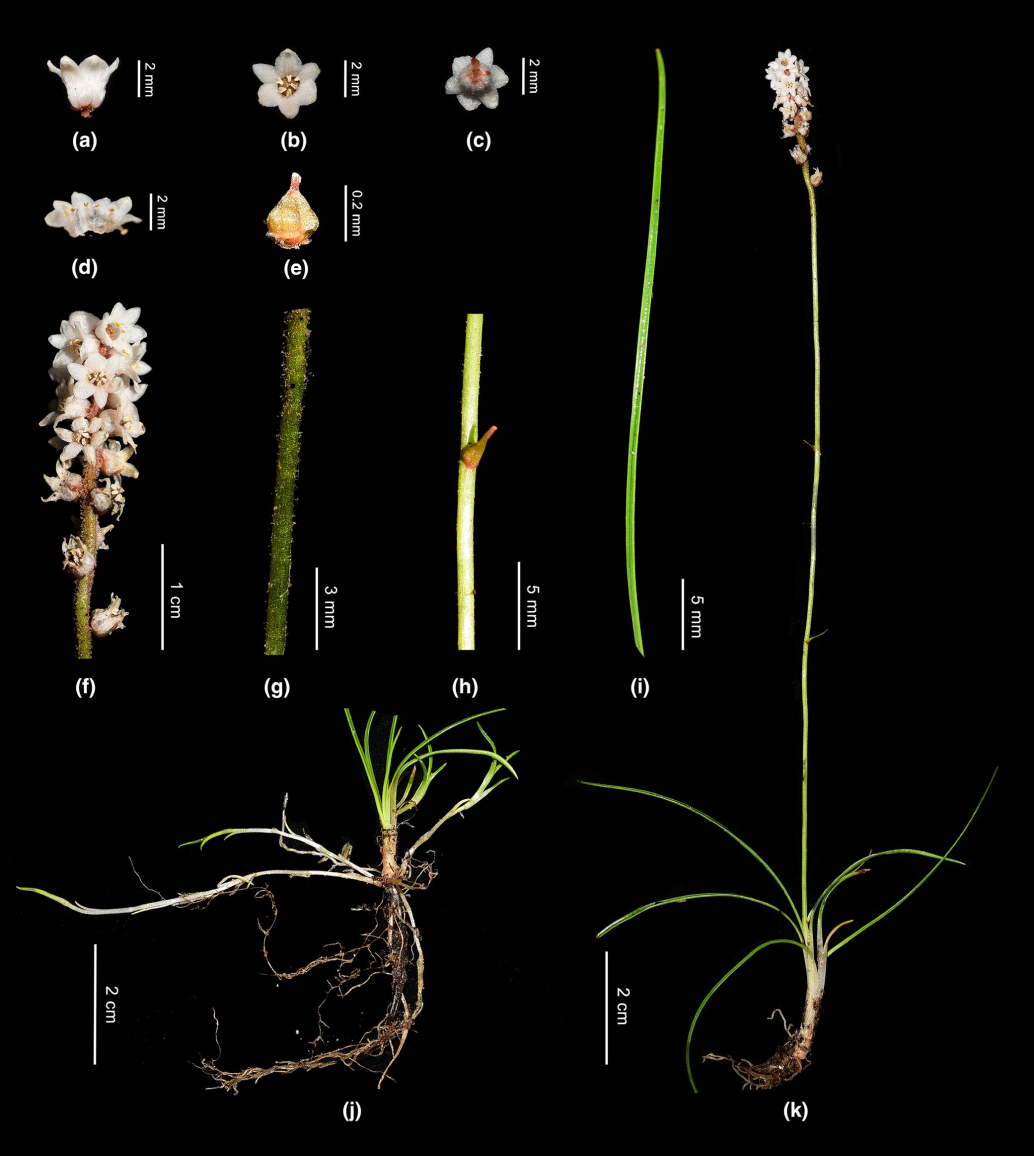
*Aletris medogensis*. Lateral (a), front (b), and back (c) views of the flower. (d) Stamens. (e) Ovary and stigma. (f) Inflorescence. (g) Scape. (h) Bracteate leaf. (i) Leaf. (k) Roots. (l) Plant.

Perennial herb, tillering with developing creeping stolons. Roots are usually fibrous. Leaves in basal rosette, occasionally, leaf primordia split from the basal region, giving rise to new leaves, linear, 2.5–9 cm × 0.45–0.9 mm, apex obtuse, glabrous. Scape 3.5–16 cm × 0.7–1 mm, glandular are sparse near the inflorescence and progressively become glabrous toward the lower part, bract‐like leaves 0.2–0.6 cm long in the middle and lower part, lanceolate or ovate‐lanceolate, apex acute. Raceme 1.5–2.5 cm, densely 10–14‐flowered, pedicels extremely short, rachis densely glandular hairs; bract and bracteole lanceolate, 2–4 mm, borne at the apex of the pedicel, shorter than the flower. Perianth white, glabrous, split to the middle; tube campanulate; lobes recurved or erect, ovate, apex obtuse to rounded, 1.2–2 × 0.8–1.5 mm. Filaments of stamens adnate to perianth, 0.5 mm, anthers elliptical, 0.5 mm, the style abruptly constricted into a short style, stigma not or only slightly thickened, capitate. Ovary ovate, ridged. Fruits capsular, 3‐locular (Figures 9 and 10).

### 6.4 Phenology

Flowering from June to July, fruiting from August to September.

### 6.5 Etymology

Located in southeastern Xizang Autonomous Region, Medog (Pinyin spelling “motuo”) County is one of the biodiversity hotspots in China, which has rich plant diversity in the Eastern Himalaya (Qiu et al. 2022). The new species, *Aletris medogensis*, is found in this region and is named after the geographic location. Its Chinese name, mo tuo fen tiao er cai (墨脱粉条儿菜).

### 6.6 Distribution and Ecology

This new species is currently known only from the opposite bank of Hanmi to the Xiaoyandong direction in Motuo County, Nyingchi City, Xizang (Tibet), China, at an elevation of 2200–2400 m. *Aletris medogensis* grows on moss‐covered rocks along streams in moist environments.

### 6.7 Conservation Status

Currently known from a small population at the type locality, approximately 150 mature individuals have been observed. The data available for this new species are still insufficient to assess its conservation status. According to the IUCN 2023 Red List criteria (International Union for Conservation of Nature 2023), the species is classified as Data Deficient (DD). Further collection and monitoring efforts are required to enable more conclusive assessments of the species' rarity and vulnerability.

## Author Contributions

**Xiong Li:** data curation (lead), software (equal), visualization (equal), writing – original draft (lead), writing – review and editing (lead). **Yong‐Ling Qiu:** data curation (equal), software (equal), writing – original draft (equal), writing – review and editing (supporting). **Jiang‐Tao Li:** investigation (equal), writing – review and editing (supporting). **Bo Xu:** writing – original draft (supporting), writing – review and editing (supporting). **Qi Yu:** writing – review and editing (supporting). **Wen‐Bin Ju:** investigation (lead), project administration (lead), resources (lead), writing – review and editing (equal).

## Funding

This work was supported by the Science and Technology Major Project of Xizang (XZ202501ZY05151), the Special Investigation and Monitoring of the Yarlung Zangbo Grand Canyon National Nature Reserve in Xizang (GZFCG202314256), and the Second Tibetan Plateau Scientific Expedition and Research (STEP) program (2024QZKK0200).

## Ethics Statement

This study's material collections and experimental research followed the relevant institutional, national, and international guidelines and legislation.

## Consent

The authors have nothing to report.

## Conflicts of Interest

The authors declare no conflicts of interest.

## Supporting information


**Appendix S1–S2:** ece372654‐sup‐0001‐AppendixS1‐S2.docx.

**Figures S1–S2:** ece372654‐sup‐0002‐FigureS1‐S2.docx.

**Tables S1–S7:** ece372654‐sup‐0003‐TableS1‐S7.xlsx.

## Data Availability

The chloroplast genome of *Aletris medogensis* has been deposited into the National Center for Biotechnology Information (NCBI) database with accession number PV472299.

## References

[ece372654-bib-0001] Adolf, E. 1905. Botanische Jahrbücher für Systematik, Pflanzengeschichte und Pflanzengeographie. Vol. 36. Forgotten Books.

[ece372654-bib-0002] Akahori, A. , F. Yasuda , and T. Okanishi . 1971. “Steroidal Sapogenins of *Aletris spicata* (Thunb.) Franchet.” Chemical & Pharmaceutical Bulletin 19: 2409–2411.

[ece372654-bib-0003] Ambrose, J. D. 1980. “A Re‐Evaluation of the Melanthioideae (Liliaceae) Using Numerical Analyses.” In Pelaloid Monocotyledons. Linnean Society Symposium Series, edited by C. D. Brickell and M. Gregory , vol. 8, 65–82. Academic Press.

[ece372654-bib-0004] Amiryousefi, A. , J. Hyvonen , and P. Poczai . 2018. “Irscope: An Online Program to Visualize the Junction Sites of Chloroplast Genomes.” Bioinformatics 34, no. 17: 3030–3031. 29659705 10.1093/bioinformatics/bty220

[ece372654-bib-0005] Barthet, M. M. , and K. W. Hilu . 2007. “Expression of Matk: Functional and Evolutionary Implications.” American Journal of Botany 94, no. 8: 1402–1412. 21636508 10.3732/ajb.94.8.1402

[ece372654-bib-0006] Beier, S. , T. Thiel , T. Munch , U. Scholz , and M. Mascher . 2017. “Misa‐Web: A Web Server for Microsatellite Prediction.” Bioinformatics 33, no. 16: 2583–2585. 28398459 10.1093/bioinformatics/btx198 PMC5870701

[ece372654-bib-0007] Brotherus, V. F. , and H. R. E. Handel‐Mazzetti . 1936. Symbolae Sinicae: Botanische Ergebnisse Der Expedition Der Akademie Der Wissenschaften in Wein Nach Südwest‐China, 1914–1918. Vol. 7. Akademie der Wissenschaften.

[ece372654-bib-0008] Caddick, L. R. , P. J. Rudall , P. Wilkin , T. A. J. Hedderson , and M. W. Chase . 2002. “Phylogenetics of Dioscoreales Based on Combined Analyses of Morphological and Molecular Data.” Botanical Journal of the Linnean Society 138, no. 2: 123–144.

[ece372654-bib-0009] Castresana, J. 2000. “Selection of Conserved Blocks From Multiple Alignments for Their Use in Phylogenetic Analysis.” Molecular Biology and Evolution 17, no. 4: 540–552. 10742046 10.1093/oxfordjournals.molbev.a026334

[ece372654-bib-0010] Challinor, V. L. , R. C. Johnston , P. V. Bernhardt , R. P. Lehmann , E. H. Krenske , and J. J. De Voss . 2015. “Biosynthetic Insights Provided by Unusual Sesterterpenes From the Medicinal Herb *Aletris farinosa* .” Chemical Science 6, no. 10: 5740–5745. 29081941 10.1039/c5sc02056e PMC5633834

[ece372654-bib-0011] Chen, S. , Y. Zhou , Y. Chen , and J. Gu . 2018. “Fastp: An Ultra‐Fast All‐in‐One Fastq Preprocessor.” Bioinformatics 34, no. 17: i884–i890. 30423086 10.1093/bioinformatics/bty560 PMC6129281

[ece372654-bib-0012] Chiba, Y. , K. Mineta , M. Y. Hirai , et al. 2013. “Changes in mRNA Stability Associated With Cold Stress in *Arabidopsis* Cells.” Plant & Cell Physiology 54, no. 2: 180–194. 23220693 10.1093/pcp/pcs164

[ece372654-bib-0013] Cvijovic, I. , B. H. Good , and M. M. Desai . 2018. “The Effect of Strong Purifying Selection on Genetic Diversity.” Genetics 209, no. 4: 1235–1278. 29844134 10.1534/genetics.118.301058 PMC6063222

[ece372654-bib-0014] Daniell, H. , C. S. Lin , M. Yu , and W. J. Chang . 2016. “Chloroplast Genomes: Diversity, Evolution, and Applications in Genetic Engineering.” Genome Biology 17, no. 1: 134. 27339192 10.1186/s13059-016-1004-2 PMC4918201

[ece372654-bib-0015] Darriba, D. , G. L. Taboada , R. Doallo , and D. Posada . 2012. “Jmodeltest 2: More Models, New Heuristics and Parallel Computing.” Nature Methods 9, no. 8: 772. 10.1038/nmeth.2109 PMC4594756 22847109

[ece372654-bib-0016] Dong, W. L. , R. N. Wang , N. Y. Zhang , W. B. Fan , M. F. Fang , and Z. H. Li . 2018. “Molecular Evolution of Chloroplast Genomes of Orchid Species: Insights Into Phylogenetic Relationship and Adaptive Evolution.” International Journal of Molecular Sciences 19, no. 3: 716. 29498674 10.3390/ijms19030716 PMC5877577

[ece372654-bib-0017] Dong, W. P. , C. Xu , C. H. Li , et al. 2015. “ *Ycf1*, the Most Promising Plastid DNA Barcode of Land Plants.” Scientific Reports 5: 8348. 25672218 10.1038/srep08348 PMC4325322

[ece372654-bib-0018] Dopp, I. J. , X. Yang , and S. A. Mackenzie . 2021. “A New Take on Organelle‐Mediated Stress Sensing in Plants.” New Phytologist 230, no. 6: 2148–2153. 33704791 10.1111/nph.17333 PMC8214450

[ece372654-bib-0019] Feng, Y. , X. F. Gao , J. Y. Zhang , et al. 2022. “Complete Chloroplast Genomes Provide Insights Into Evolution and Phylogeny of *Campylotropis* (Fabaceae).” Frontiers in Plant Science 13: 895543. 35665174 10.3389/fpls.2022.895543 PMC9158520

[ece372654-bib-0020] Fuse, S. , N. S. Lee , and M. N. Tamura . 2012. “Biosystematic Studies on the Family Nartheciaceae (Dioscoreales) I. Phylogenetic Relationships, Character Evolution and Taxonomic re‐Examination.” Plant Systematics and Evolution 298, no. 8: 1575–1584.

[ece372654-bib-0021] Greiner, S. , P. Lehwark , and R. Bock . 2019. “Organellargenomedraw (Ogdraw) Version 1.3.1: Expanded Toolkit for the Graphical Visualization of Organellar Genomes.” Nucleic Acids Research 47, no. W1: W59–W64. 30949694 10.1093/nar/gkz238 PMC6602502

[ece372654-bib-0022] Huang, L. J. , H. X. Yu , Z. Wang , and W. B. Xu . 2024. “Cpstools: A Package for Analyzing Chloroplast Genome Sequences.” iMetaOmics 1, no. 2: e25. 10.1002/imo2.25 PMC12806284 41676121

[ece372654-bib-0023] International Union for Conservation of Nature . 2023. The IUCN Red List of Threatened Species. Version 2023. IUCN.

[ece372654-bib-0024] Jin, J. J. , W. B. Yu , J. B. Yang , et al. 2020. “Getorganelle: A Fast and Versatile Toolkit for Accurate De Novo Assembly of Organelle Genomes.” Genome Biology 21, no. 1: 241. 32912315 10.1186/s13059-020-02154-5 PMC7488116

[ece372654-bib-0025] Katoh, K. , and D. M. Standley . 2013. “Mafft Multiple Sequence Alignment Software Version 7: Improvements in Performance and Usability.” Molecular Biology and Evolution 30, no. 4: 772–780. 23329690 10.1093/molbev/mst010 PMC3603318

[ece372654-bib-0026] Keller, J. , M. Rousseau‐Gueutin , G. E. Martin , et al. 2017. “The Evolutionary Fate of the Chloroplast and Nuclear *rps16* Genes as Revealed Through the Sequencing and Comparative Analyses of Four Novel Legume Chloroplast Genomes From Lupinus.” DNA Research 24, no. 4: 343–358. 28338826 10.1093/dnares/dsx006 PMC5737547

[ece372654-bib-0027] Kim, K. J. , and H. L. Lee . 2005. “Widespread Occurrence of Small Inversions in the Chloroplast Genomes of Land Plants.” Molecules and Cells 19, no. 1: 104–113. 15750347

[ece372654-bib-0028] Krasileva, K. V. , H. A. Vasquez‐Gross , T. Howell , et al. 2017. “Uncovering Hidden Variation in Polyploid Wheat.” Proceedings of the National Academy of Sciences of the United States of America 114, no. 6: E913–E921. 28096351 10.1073/pnas.1619268114 PMC5307431

[ece372654-bib-0029] Kurtz, S. , J. V. Choudhuri , E. Ohlebusch , C. Schleiermacher , J. Stoye , and R. Giegerich . 2001. “Reputer: The Manifold Applications of Repeat Analysis on a Genomic Scale.” Nucleic Acids Research 29, no. 22: 4633–4642. 11713313 10.1093/nar/29.22.4633 PMC92531

[ece372654-bib-0030] Li, C. Y. , Y. L. Zhao , Z. G. Xu , G. Y. Yang , J. Peng , and X. Y. Peng . 2020. “Initial Characterization of the Chloroplast Genome of *Vicia sepium*, an Important Wild Resource Plant, and Related Inferences About Its Evolution.” Frontiers in Genetics 11: 73. 32153639 10.3389/fgene.2020.00073 PMC7044246

[ece372654-bib-0031] Li, L. Z. , M. H. Wang , J. B. Sun , and J. Y. Liang . 2014. “Flavonoids and Other Constituents From *Aletris spicata* and Their Chemotaxonomic Significance.” Natural Product Research 28, no. 15: 1214–1217. 24896299 10.1080/14786419.2014.921918

[ece372654-bib-0032] Li, X. , L. S. Jiang , H. N. Deng , et al. 2025. “High‐Resolution Genome Assembly and Population Genetic Study of the Endangered Maple *Acer pentaphyllum* (Sapindaceae): Implications for Conservation Strategies.” Horticulture Research 12, no. 4: uhae357. 40066161 10.1093/hr/uhae357 PMC11891484

[ece372654-bib-0033] Liang, S. Y. , and N. J. Turland . 2000. “Aletris.” In Flora of China, edited by Z. Wu and P. Raven , vol. 24, 77–82. Science Press.

[ece372654-bib-0034] Lin, C. P. , C. S. Wu , Y. Y. Huang , and S. M. Chaw . 2012. “The Complete Chloroplast Genome of *Ginkgo biloba* Reveals the Mechanism of Inverted Repeat Contraction.” Genome Biology and Evolution 4, no. 3: 374–381. 22403032 10.1093/gbe/evs021 PMC3318433

[ece372654-bib-0035] Linnaeus, C. 1753. Species Plantarum. Vol. 1. Impensis Laurentii Salvii.

[ece372654-bib-0036] Liu, Q. P. , and Q. Z. Xue . 2005. “Comparative Studies on Codon Usage Pattern of Chloroplasts and Their Host Nuclear Genes in Four Plant Species.” Journal of Genetics 84, no. 1: 55–62. 15876584 10.1007/BF02715890

[ece372654-bib-0037] Lu, R. S. , P. Li , and Y. X. Qiu . 2016. “The Complete Chloroplast Genomes of Three *Cardiocrinum* (Liliaceae) Species: Comparative Genomic and Phylogenetic Analyses.” Frontiers in Plant Science 7: 2054. 28119727 10.3389/fpls.2016.02054 PMC5222849

[ece372654-bib-0038] Mayor, C. , M. Brudno , J. R. Schwartz , et al. 2000. “Vista: Visualizing Global DNA Sequence Alignments of Arbitrary Length.” Bioinformatics 16, no. 11: 1046–1047. 11159318 10.1093/bioinformatics/16.11.1046

[ece372654-bib-0039] Merckx, V. , P. Schols , K. Geuten , S. Huysmans , and E. Smets . 2008. “Phylogenetic Relationships in Nartheciaceae (Dioscoreales), With Focus on Pollen and Orbicule Morphology.” Belgian Journal of Botany 141: 64–77.

[ece372654-bib-0040] Messer, P. W. , and D. A. Petrov . 2013. “Population Genomics of Rapid Adaptation by Soft Selective Sweeps.” Trends in Ecology & Evolution 28, no. 11: 659–669. 24075201 10.1016/j.tree.2013.08.003 PMC3834262

[ece372654-bib-0041] Moseley, R. C. , R. Mewalal , F. Motta , G. A. Tuskan , S. Haase , and X. H. Yang . 2018. “Conservation and Diversification of Circadian Rhythmicity Between a Model Crassulacean Acid Metabolism Plant Kalanchoë Fedtschenkoi and a Model C3 Photosynthesis Plant *Arabidopsis thaliana* .” Frontiers in Plant Science 9: 1757. 30546378 10.3389/fpls.2018.01757 PMC6279919

[ece372654-bib-0042] Nguyen, L. T. , H. A. Schmidt , A. von Haeseler , and B. Q. Minh . 2015. “Iq‐Tree: A Fast and Effective Stochastic Algorithm for Estimating Maximum‐Likelihood Phylogenies.” Molecular Biology and Evolution 32, no. 1: 268–274. 25371430 10.1093/molbev/msu300 PMC4271533

[ece372654-bib-0043] Nong, Y. , K. D. Lai , Y. R. Qin , et al. 2024. “ *Aletris guangxiensis* (Nartheciaceae), a New Species From Guangxi, China.” PhytoKeys 237: 79–89. 38282985 10.3897/phytokeys.237.115037 PMC10819618

[ece372654-bib-0044] Ogawa, T. , E. Marco , and M. I. Orus . 1994. “A Gene (ccma) Required for Carboxysome Formation in the Cyanobacterium *Synechocystis* sp Strain PCC6803.” Journal of Bacteriology 176, no. 8: 2374–2378. 8157606 10.1128/jb.176.8.2374-2378.1994 PMC205361

[ece372654-bib-0045] Oliver, K. R. , J. A. McComb , and W. K. Greene . 2013. “Transposable Elements: Powerful Contributors to Angiosperm Evolution and Diversity.” Genome Biology and Evolution 5, no. 10: 1886–1901. 24065734 10.1093/gbe/evt141 PMC3814199

[ece372654-bib-0046] Porebski, S. , L. G. Bailey , and B. R. Baum . 1997. “Modification of a CTAB DNA Extraction Protocol for Plants Containing High Polysaccharide and Polyphenol Components.” Plant Molecular Biology Reporter 15, no. 1: 8–15.

[ece372654-bib-0047] Qiu, Y. L. , K. W. Xu , W. B. Ju , W. L. Zhao , and L. Zhang . 2022. “ *Hymenasplenium tholiformis* (Aspleniaceae), a New Fern Species From Southeastern Xizang, China Based on Morphological and Molecular Evidence.” PhytoKeys 204: 43–56. 36760613 10.3897/phytokeys.204.85746 PMC9848882

[ece372654-bib-0048] Qu, X. J. , M. J. Moore , D. Z. Li , and T. S. Yi . 2019. “PGA: A Software Package for Rapid, Accurate, and Flexible Batch Annotation of Plastomes.” Plant Methods 15: 50. 31139240 10.1186/s13007-019-0435-7 PMC6528300

[ece372654-bib-0049] Ramundo, S. , M. Rahire , O. Schaad , and J. D. Rochaix . 2013. “Repression of Essential Chloroplast Genes Reveals New Signaling Pathways and Regulatory Feedback Loops in.” Plant Cell 25, no. 1: 167–186. 23292734 10.1105/tpc.112.103051 PMC3584532

[ece372654-bib-0050] Redwan, R. M. , A. Saidin , and S. V. Kumar . 2015. “Complete Chloroplast Genome Sequence of MD‐2 Pineapple and Its Comparative Analysis Among Nine Other Plants From the Subclass Commelinidae.” BMC Plant Biology 15: 196. 26264372 10.1186/s12870-015-0587-1 PMC4534033

[ece372654-bib-0051] Ronquist, F. , M. Teslenko , P. van der Mark , et al. 2012. “Mrbayes 3.2: Efficient Bayesian Phylogenetic Inference and Model Choice Across a Large Model Space.” Systematic Biology 61, no. 3: 539–542. 22357727 10.1093/sysbio/sys029 PMC3329765

[ece372654-bib-0052] Rui, L. , S. Q. Yang , X. H. Zhou , and W. Wang . 2025. “The Important Role of Chloroplasts in Plant Immunity.” Plant Communications 6, no. 8: 101420. 40534128 10.1016/j.xplc.2025.101420 PMC12365846

[ece372654-bib-0053] Sabir, J. , E. Schwarz , N. Ellison , et al. 2014. “Evolutionary and Biotechnology Implications of Plastid Genome Variation in the Inverted‐Repeat‐Lacking Clade of Legumes.” Plant Biotechnology Journal 12, no. 6: 743–754. 24618204 10.1111/pbi.12179

[ece372654-bib-0054] Schneider, C. A. , W. S. Rasband , and K. W. Eliceiri . 2012. “NIH Image to ImageJ: 25 Years of Image Analysis.” Nature Methods 9, no. 7: 671–675. 22930834 10.1038/nmeth.2089 PMC5554542

[ece372654-bib-0055] Shi, Y. J. , J. L. Huang , X. Q. Wan , J. L. Shi , Z. Chen , and W. Zeng . 2025. “The Population Chloroplast Genomes of *Populus* Reveal the Phylogenetic Relationship Between Three New Taxa of Sect. *Leucoides* and Their Parents.” BMC Genomics 26, no. 1: 156. 39962394 10.1186/s12864-024-11099-z PMC11834202

[ece372654-bib-0056] Smarda, P. , P. Bures , L. Horová , et al. 2014. “Ecological and Evolutionary Significance of Genomic Gc Content Diversity in Monocots.” Proceedings of the National Academy of Sciences of the United States of America 111, no. 39: E4096–E4102. 25225383 10.1073/pnas.1321152111 PMC4191780

[ece372654-bib-0057] Sugiura, M. 1992. “The Chloroplast Genome.” Plant Molecular Biology 19, no. 1: 149–168. 1600166 10.1007/BF00015612

[ece372654-bib-0058] Tsudzuki, J. , K. Nakashima , T. Tsudzuki , et al. 1992. “Chloroplast DNA of Black Pine Retains a Residual Inverted Repeat Lacking Rrna Genes: Nucleotide Sequences of trnq, trnk, psba, trni and trnh and the Absence of rps16.” Molecular & General Genetics 232, no. 2: 206–214. 1557027 10.1007/BF00279998

[ece372654-bib-0059] Wang, D. , Y. Zhang , Z. Zhang , J. Zhu , and J. Yu . 2010. “Kaks_Calculator 2.0: A Toolkit Incorporating Gamma‐Series Methods and Sliding Window Strategies.” Genomics, Proteomics & Bioinformatics 8, no. 1: 77–80. 10.1016/S1672-0229(10)60008-3 PMC5054116 20451164

[ece372654-bib-0060] Wang, F. J. , and J. Tang . 1978. Flora of China. Vol. 15, 172. Science Press.

[ece372654-bib-0061] Wei, P. , Y. Z. Li , M. Ke , Y. R. Hou , A. Aikebaier , and Z. N. Wu . 2024. “Complete Chloroplast Genome of *Krascheninnikovia ewersmanniana*: Comparative and Phylogenetic Analysis.” Genes 15, no. 5: 546. 38790176 10.3390/genes15050546 PMC11121282

[ece372654-bib-0062] Wicker, T. , F. Sabot , A. Hua‐Van , et al. 2007. “A Unified Classification System for Eukaryotic Transposable Elements.” Nature Reviews. Genetics 8, no. 12: 973–982. 10.1038/nrg2165 17984973

[ece372654-bib-0063] Wu, Z. Y. , A. M. Lu , Y. C. Tang , Z. D. Chen , and D. Z. Li . 2003. The Families and Genera of Angiosperms in China: A Comprehensive Analysis, 252. Science Press.

[ece372654-bib-0064] Xie, Z. Y. , and S. Merchant . 1996. “The Plastid‐Encoded *ccsa* Gene Is Required for Heme Attachment to Chloroplast C‐Type Cytochromes.” Journal of Biological Chemistry 271, no. 9: 4632–4639. 8617725 10.1074/jbc.271.9.4632

[ece372654-bib-0065] Xu, J. C. , R. E. Grumbine , A. Shrestha , et al. 2009. “The Melting Himalayas: Cascading Effects of Climate Change on Water, Biodiversity, and Livelihoods.” Conservation Biology 23, no. 3: 520–530. 22748090 10.1111/j.1523-1739.2009.01237.x

[ece372654-bib-0066] Xu, S. J. , K. Teng , H. Zhang , et al. 2023. “Chloroplast Genomes of Four Species: Long Repetitive Sequences Trigger Dramatic Changes in Chloroplast Genome Structure.” Frontiers in Plant Science 14: 1100876. 36778700 10.3389/fpls.2023.1100876 PMC9911286

[ece372654-bib-0067] Yang, J. , D. D. Liu , T. Chen , J. Liu , X. P. Ma , and Z. H. Wang . 2024. “Extraction Process Optimization and Antioxidant, Anti‐Inflammatory Activity of Total Flavonoids From *Aletris spicata* (Thunb.) Franch.” Science and Technology of Food Industry 45, no. 2: 192–200.

[ece372654-bib-0068] Zhang, K. R. , and Z. Q. Yan . 2023. “Editorial: The Impacts of Climate Change and Human Activities on the Structure and Function of Wetland/Grassland Ecosystems.” Frontiers in Ecology and Evolution 11: 1296677.

[ece372654-bib-0069] Zhang, Y. , A. H. Zhang , X. M. Li , and C. M. Lu . 2020. “The Role of Chloroplast Gene Expression in Plant Responses to Environmental Stress.” International Journal of Molecular Sciences 21, no. 17: 6082. 32846932 10.3390/ijms21176082 PMC7503970

[ece372654-bib-0070] Zhang, Z. , J. Xiao , J. Wu , et al. 2012. “Paraat: A Parallel Tool for Constructing Multiple Protein‐Coding DNA Alignments.” Biochemical and Biophysical Research Communications 419, no. 4: 779–781. 22390928 10.1016/j.bbrc.2012.02.101

[ece372654-bib-0071] Zhang, Z. R. , X. Yang , W. Y. Li , Y. Q. Peng , and J. Gao . 2022. “Comparative Chloroplast Genome Analysis of *Ficus* (Moraceae): Insight Into Adaptive Evolution and Mutational Hotspot Regions.” Frontiers in Plant Science 13: 965,335. 10.3389/fpls.2022.965335 PMC9521400 36186045

[ece372654-bib-0072] Zhao, B. , J. J. Li , R. W. Yuan , and S. Z. Mao . 2017. “Adaptive Evolution of the *Rbcl* Gene in the Genus Rheum (Polygonaceae).” Biotechnology & Biotechnological Equipment 31, no. 3: 493–498.

[ece372654-bib-0073] Zhao, C. C. , Y. Y. Wang , K. X. Chan , et al. 2019. “Evolution of Chloroplast Retrograde Signaling Facilitates Green Plant Adaptation to Land.” Proceedings of the National Academy of Sciences of the United States of America 116, no. 11: 5015–5020. 30804180 10.1073/pnas.1812092116 PMC6421419

[ece372654-bib-0074] Zhao, Y. M. , W. Wang , and S. R. Zhang . 2012. “Delimitation and Phylogeny of *Aletris* (Nartheciaceae) With Implications for Perianth Evolution.” Journal of Systematics and Evolution 50, no. 2: 135–145.

[ece372654-bib-0075] Zoschke, R. , and R. Bock . 2018. “Chloroplast Translation: Structural and Functional Organization, Operational Control, and Regulation.” Plant Cell 30, no. 4: 745–770. 29610211 10.1105/tpc.18.00016 PMC5969280

