# Supplementary material for: Integrating Morphology and Chloroplast Genomics: A New East Asian Species of Aletris (Nartheciaceae) With Insights Into Regional Phylogeny and Evolution
Source: Ecol Evol. 2026 Jan 5;16(1):e72654. doi: 10.1002/ece3.72654 (PMC12771657; doi:10.1002/ece3.72654)
Supplement: Supplementary file 1 — Appendix S1–S2: ece372654‐sup‐0001‐AppendixS1‐S2.docx. [file ECE3-16-e72654-s003.docx]

## Appendix S1 *Characteristic_statistic.py*

| #!/usr/bin/python3 # usage: python Characteristic_statistic.py info.txt ./fastas/ ./gbs/ > statistic.txt  *import* sys, subprocess *from* Bio *import* SeqIO  *def* Calculate_length_gc(*regions*, *fasta_file*):  length = 0  gc_sum = 0  region_list = *regions*.split(',')  *for* region *in* region_list:  start, end = map(int, region.split('-'))  length += end - start + 1  cmd = "seqkit subseq -r " + str(start) + ":" + str(end) + " " + *fasta_file* + \  " \| seqkit fx2tab -l -g -n -i -H \| grep -v '#' \| awk '{print$NF}' "  output = float(subprocess.check_output(cmd, shell=*True*, text=*True*)) # 注意不要用int，不然会报错  gc_sum += output  gc = round(float(gc_sum) / (len(*regions*.split(','))),2) # 保留两位小数  *return* length, gc  *def* Count_gene_num(*gb_file*):  protein_count = 0  trna_count = 0  rrna_count = 0  *for* record *in* SeqIO.parse(*gb_file*, "genbank"):  *for* feature *in* record.features:  *if* feature.type == "CDS":  protein_count += 1  *elif* feature.type == "tRNA":  trna_count += 1  *elif* feature.type == "rRNA":  rrna_count += 1  *return* protein_count, trna_count, rrna_count  *with* open (sys.argv[1], 'r', encoding='utf-8') *as* fp:  *for* line *in* fp :  infos = line.strip().split('\t')  species = infos[0].split('.gb')[0]  fasta_file = sys.argv[2] + species+'.fasta'  gb_file = sys.argv[3] + species+'.gb'  LSC_length, LSC_gc = Calculate_length_gc(infos[1].split(':')[1], fasta_file)  SSC_length, SSC_gc = Calculate_length_gc(infos[3].split(':')[1], fasta_file)  IRa_length, IRa_gc = Calculate_length_gc(infos[4].split(':')[1], fasta_file)  IRb_length, IRb_gc= Calculate_length_gc(infos[2].split(':')[1], fasta_file)  Total_length = LSC_length + SSC_length + IRa_length + IRb_length  cmd = "seqkit fx2tab -l -g -n -i -H " + fasta_file+ " \| grep -v '#' \| awk '{print$NF}' "  Total_gc = round(float(subprocess.check_output(cmd, shell=*True*, text=*True*)),2)  cds, tRNA, rRNA = Count_gene_num(gb_file)  Total_gene = cds + tRNA + rRNA  *if* IRa_length == IRb_length:  print(f"{species}\t{Total_length}\t{LSC_length}\t{SSC_length}\t{IRa_length}\t{IRb_length}\t"  f"{Total_gc}\t{LSC_gc}\t{SSC_gc}\t{IRa_gc}\t{IRb_gc}\t{Total_gene}\t{cds}\t{tRNA}\t{rRNA}")  *else*:  print(f"{species}\tERROR") |
| --- |

## Appendix S2 *Hypervariable_region_idenfied.py*

| #!/usr/bin/python3  # usage: python Hypervariable_region_idenfied.py ./14cp_diversity.csv ./14cp_mafft.fasta  *import* pandas *as* pd *from* Bio *import* AlignIO *from* itertools *import* combinations *from* collections *import* Counter  *def* load_window_data(*file_path*):  """加载窗口数据文件"""  df = pd.read_csv(*file_path*, sep=",")  df.columns = ['win_start', 'length', 'num_snp', 'num_missing', 'num_indel', 'num_svs', 'num_pip',  'theta', 'pi']  df['win_end'] = df['win_start'] + df['length'] - 1 #需要减1才能代表真实位置  *return* df  *def* merge_high_var_regions(*df*, *pi_quantile*, *snp_threshold*):  """筛选并合并高变区域"""  # 计算阈值  pi_threshold = *df*['pi'].quantile(*pi_quantile*)  # 筛选高变窗口  high_var = *df*[  (*df*['pi'] > pi_threshold) &  (*df*['num_snp'] > *snp_threshold*)  ].sort_values('win_start').reset_index(drop=*True*)  *if* high_var.empty:  *return* pd.DataFrame()   # 合并相邻或重叠区域  merged = []  current_start = high_var.iloc[0]['win_start']  current_end = high_var.iloc[0]['win_end']   *for* idx *in* range(1, len(high_var)):  row = high_var.iloc[idx]  *if* row['win_start'] <= current_end + 100: #可以增加数值来降低合并条件  current_end = max(current_end, row['win_end'])  *else*:  merged.append({'Start': current_start, 'End': current_end})  current_start = row['win_start']  current_end = row['win_end']   merged.append({'Start': current_start, 'End': current_end})   *return* pd.DataFrame(merged)  *def* calculate_region_metrics(*alignment*, *start*, *end*):  """计算指定区域的变异指标"""  # 转换为0-based坐标  start_0 = int(*start* - 1)  end_0 = int(*end*)   # 提取区域比对数据  region_aln = *alignment*[:, start_0:end_0]   # 初始化统计量  n_seqs = len(region_aln)  total_sites = int(*end* - *start* + 1)  missing_cutoff = 0.5  num_missing = 0  num_snps = 0  num_indel = 0  num_svs = 0  num_pip = 0  total_pi = 0   # 遍历每个位点  *for* i *in* range(region_aln.get_alignment_length()):  base_list = list(region_aln[:, i])  filtered_bases = []  # 处理缺失数据  *if* '-' *in* base_list:  missing_proportion = base_list.count('-') / len(base_list)  filtered_bases = [b *for* b *in* base_list *if* b != '-']  *if* missing_proportion >= missing_cutoff:  num_missing += 1  *continue  else*:  num_indel += 1  *else*:  filtered_bases = base_list   # 跳过无效位点  *if* len(filtered_bases) < 2:  *continue* # SVS和PIP判断逻辑（互斥）  counts = Counter(filtered_bases)  unique_bases = len(counts)   # 判断SVS（仅两种碱基且其中一个出现一次）  *if* unique_bases == 2 *and* 1 *in* counts.values():  num_svs += 1   # 判断PIP（至少两种碱基，且所有碱基出现次数≥2）  pip_condition = (  unique_bases >= 2 *and* all(count >= 2 *for* count *in* counts.values())  )  *if* pip_condition:  num_pip += 1   # 原始SNP和PI计算逻辑  pairs = combinations(filtered_bases, 2)  num_diff = sum(1 *for* a, b *in* pairs *if* a != b)  total_pairs = len(list(combinations(filtered_bases, 2)))   *if* total_pairs > 0:  site_pi = num_diff / total_pairs  total_pi += site_pi  *if* num_diff > 0:  num_snps += 1 # SNP数量 >= SVS数量 + PIP数量  # 最终统计量计算  effective_length = total_sites - num_missing  pi = total_pi / effective_length *if* effective_length > 0 *else* 0   *return* {  'SNP': num_snps,  'SVS': num_svs,  'PIP': num_pip,  'PI': round(pi, 6)  }  *def* process_fasta_regions(*fasta_path*, *regions_df*):  """处理FASTA文件计算区域指标"""  alignment = AlignIO.read(*fasta_path*, "fasta")  results = []  *for* _, row *in regions_df*.iterrows():  metrics = calculate_region_metrics(alignment, row['Start'], row['End'])  results.append({  'Start': row['Start'],  'End': row['End'],  'Length': row['End'] - row['Start'] + 1,  **metrics  })  *return* pd.DataFrame(results)   # 主流程 *if* __name__ == "__main__":  # 1. 加载窗口数据  windows_df = load_window_data("14cp_win_diversity.csv")   # 2. 合并高变区域  merged_regions = merge_high_var_regions(windows_df, 0.95, 25) #设置pi和snp阈值   *if not* merged_regions.empty:  # 3. 从FASTA重新计算指标  merged_regions = process_fasta_regions("14cp_Aletris_mafft.fasta", merged_regions)   # 4. 输出结果  merged_regions.to_csv("Hypervariable_regions_metrics.csv", index=*False*)  *else*:  print("未检测到高变区域") |
| --- |
