# Supplementary material for: Integrating Morphology and Chloroplast Genomics: A New East Asian Species of Aletris (Nartheciaceae) With Insights Into Regional Phylogeny and Evolution
Source: Ecol Evol. 2026 Jan 5;16(1):e72654. doi: 10.1002/ece3.72654 (PMC12771657; doi:10.1002/ece3.72654)
Supplement: Supplementary file 2 — Figures S1–S2: ece372654‐sup‐0002‐FigureS1‐S2.docx. [file ECE3-16-e72654-s002.docx]

**Figure S1.** Holotype specimen of *Aletris medogensis.*


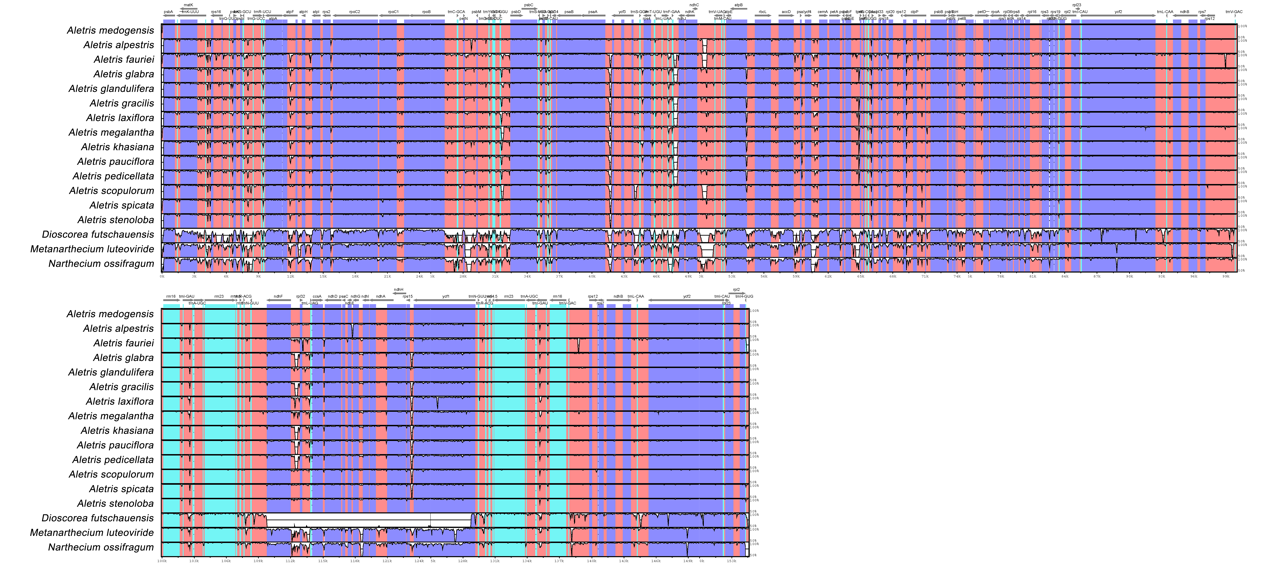


**Figure S2.** mVISTA identity plot based on the complete chloroplast genome alignments of the 17 Nartheciaceae species.
